# Heteroacene-Based Amphiphile as a Molecular Scaffold for Bioimaging Probes

**DOI:** 10.3389/fchem.2021.729125

**Published:** 2021-08-18

**Authors:** Tharindu A. Ranathunge, Mahesh Loku Yaddehige, Jordan H. Varma, Cameron Smith, Jay Nguyen, Iyanuoluwani Owolabi, Wojciech Kolodziejczyk, Nathan I. Hammer, Glake Hill, Alex Flynt, Davita L. Watkins

**Affiliations:** ^1^ Department of Chemistry and Biochemistry, University of Mississippi University, Oxford, MS, United States; ^2^ Cellular and Molecular Biology, The University of Southern Mississippi, Hattiesburg, MS, United States; ^3^ Interdisciplinary Center for Nanotoxicity, Department of Chemistry, Physics and Atmospheric Sciences, Jackson State University, Jackson, MS, United States

**Keywords:** bioimaging, Stokes-shift, heteroacenes, amphiphile, nanoparticles

## Abstract

The challenges faced with current fluorescence imaging agents have motivated us to study two nanostructures based on a hydrophobic dye, 6*H*-pyrrolo[3,2-*b*:4,5-*b*’]bis [1,4]benzothiazine (TRPZ). TRPZ is a heteroacene with a rigid, pi-conjugated structure, multiple reactive sites, and unique spectroscopic properties. Here we coupled TRPZ to a tert-butyl carbamate (BOC) protected 2,2-bis(hydroxymethyl)propanoic acid (bisMPA) dendron via azide-alkyne Huisgen cycloaddition. Deprotection of the protected amine groups on the dendron afforded a cationic terminated amphiphile, **TRPZ-bisMPA**. **TRPZ-bisMPA** was nanoprecipitated into water to obtain nanoparticles (NPs) with a hydrodynamic radius that was <150 nm. For comparison, **TRPZ-PG** was encapsulated in pluronic-F127 (Mw = 12 kD), a polymer surfactant to afford NPs almost twice as large as those formed by **TRPZ-bisMPA**. Size and stability studies confirm the suitability of the **TRPZ-bisMPA NPs** for biomedical applications. The photophysical properties of the **TRPZ-bisMPA NPs** show a quantum yield of 49%, a Stokes shift of 201 nm (0.72 eV) and a lifetime of 6.3 ns in water. Further evidence was provided by cell viability and cellular uptake studies confirming the low cytotoxicity of **TRPZ-bisMPA NPs** and their potential in bioimaging.

## Introduction

Bioimaging techniques are crucial to understanding biological processes of living systems. Among many imaging techniques, fluorescence imaging (visible to near-infrared, >400 nm) is a powerful, noninvasive method for diagnostics. It can provide excellent spatiotemporal resolution that affords the investigation of biological systems in real-time (Haustein and Schwille, 2007). In light of the aforementioned advantages, some application-based drawbacks remain in regards to the most common imaging agents and dyes (James and Gambhir, 2012). These include inadequate stability, low water solubility, and poor biocompatibility (Choi and Frangioni, 2010; Khan et al., 2019).

Inorganic hybrids such as single-wall carbon nanotubes (SWCNTs) (Jena et al., 2020) and inorganic quantum dots (QDs) (Gil et al., 2021) currently show the most promise, possessing high stability; however, these materials exhibit poor metabolism and high toxicity (Gil et al., 2021). Seeking solely organic or carbon-based alternatives, fluorescent dyes, e.g., rhodamine, (Grimm et al., 2020), fluorescein, (Ando et al., 2019), oxazine, (Vogelsang et al., 2009), have been widely adopted. However, they too exhibit unfavorable properties, specifically small Stokes shifts, which typically results in self-quenching and images with poor signal-to-noise ratios. With considerable interest in bioimaging applications, focused strategies have been in place to garner highly soluble emissive materials with low toxicity and large Stokes shifts for accurate and high-resolution images.

**FIGURE 1:**
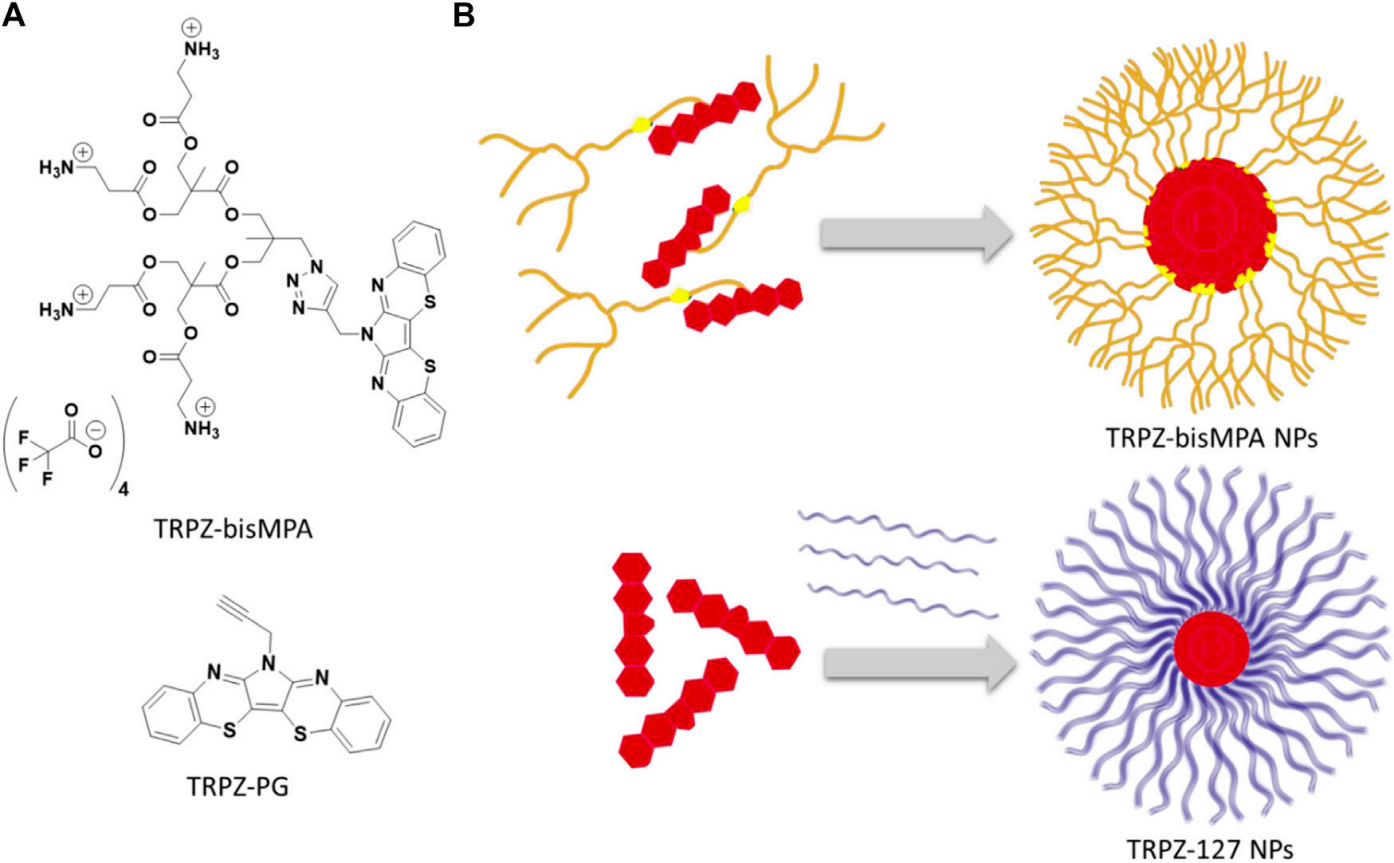
Structure of TRPZ-bisMPA **(top)** and TRPZ-PG **(bottom)** **(A)**; depiction of TRPZ-bisMPA dye-polymer amphiphile self-assembly and TRPZ-PG-Pluronic F-127 (TRPZ-127) **(B)**.

Current research efforts in bioimaging have taken a more supramolecular approach in which nanoparticles (NPs) are beginning to dominate (Mourdikoudis et al., 2018; Zhang et al., 2019; Zhang et al., 2020). These strategies have afforded biocompatible, water-soluble efficient probes where hydrophobic fluorophores undergo a structural organization that contributes to favorable photophysical properties. In this study, we designed and synthesized a heteroacene amphiphile based on 6*H*-pyrrolo[3,2-*b*:4,5-*b*’]bis[1,4]benzothiazine (TRPZ). The structures of interest are shown in Figure 1, where the hydrophobic framework of TRPZ has been modified to form a self-assembling species capable of forming stable NPs for bioimaging applications.

TRPZ is similar to pentacene and pyrene in that it possesses a conjugated structure and unique spectroscopic properties. Its structure readily forms pi-aggregates/excimers at high concentrations due to the stacking interactions of the pi-conjugated backbone resulting in a red-shift of the fluorescence and a bright green-yellow emission. Additionally, it possesses multiple reactive sites with the central pyrrole nitrogen atom offering a diverse library of derivatives with potentially unique functionalities.

Here we propargylated the central nitrogen on TRPZ (**TRPZ-PG**) and coupled it to a dendritic bisMPA alanine possessing protected amine termini via copper(I)-catalyzed azide-alkyne cycloaddition (CuAAC). Deprotection of tert-butyl carbamates (BOC) protected amine groups with trifluoroacetic acid (TFA) afforded a cationic terminated **TRPZ-bisMPA** amphiphile. In aqueous media, **TRPZ-bisMPA** is capable of self-assembling into nanoparticles (NPs) where TRPZ makes up the core and bisMPA is the corona or exterior. In parallel, **TRPZ-PG** was encapsulated in pluronic-F127 (Mw = 12 kD), a polymer surfactant, to make a second NP system, **TRPZ-127 NPs**. We report the size and photophysical properties of the two resulting NP systems. Based on these results, **TRPZ-bisMPA** was then assessed via biological studies to demonstrate TRPZ as a promising platform for designing new versatile bioimaging probes.

## Materials and Methods

Reagents and solvents were purchased from Sigma-Aldrich and used without further purification unless otherwise specified. All synthetic procedures were carried out under nitrogen atmosphere using standard Schlenk line techniques unless otherwise stated. Additional synthetic details and general procedures are given in the electronic supporting information (SI).

### Synthesis of A-MPA-4-ala

**Synthesis of (2,2,5-trimethyl-1,3-dioxan-5-yl)methanol (2):** The 2-(hydroxymethyl)-2-methylpropane-1,3-diol (10.10 g, 80.1 mmol) was stirred in 50 ml of acetone, 2,2-dimethoxypropane (DMP) (13.1 g, 126 mmol) and PTSA (0.79 g, 4.14 mmol) were added under room temperature. After the completion of the addition, the reaction mixture was stirred for 4 h. Then it was filtered through an amberlyst column, and the solvent was evaporated, and the residue was put under 60°C and full vacuum for 2 h. Then it was put under vacuum overnight to give (2) as a colorless liquid with 96% yield. ^1^H NMR (500 MHz, CDCl_3_) δ 3.67 [m, (d, s overlap J = 11.8 Hz, 2H; s, 2H)], 3.60 (d, J = 11.8 Hz, 2H), 2.55 (s, 1H), 1.44 (s, 3H), 1.40 (s, 3H), 0.83 (s, 3H).

**Synthesis of (2,2,5-trimethyl-1,3-dioxan-5-yl)methyl 4-methylbenzenesulfonate (3):** Compound (2) (10.9 g, 68.0 mmol) was dissolved in 34 ml of pyridine, and it was added dropwise to the stirred solution of p-toluenesulfonyl chloride (35.7 g, 187 mmol) in 48 ml of pyridine at 0°C under nitrogen. After the complete addition, the reaction mixture was stirred 48 h at room temperature. Then the reaction mixture was added dropwise to 100 ml of 40% ammonium chloride solution at 0°C. After complete addition, it was allowed to stir at room temperature for 2 h. Then it was filtered and washed with DI water until the pyridine smell was gone. Then the residue was dissolved in 25 ml of DCM and extracted with half saturated ammonium chloride and saturated NaCl solution. Yellow DCM solution was dried with anhydrous sodium sulfate. Then the solvent was evaporated, and the residue was placed under full vacuum for 12 h to give (3) as a yellow solid with 89% yield. ^1^H NMR (500 MHz, CDCl_3_) δ 7.86–7.79 (m, 2H), 7.38 (d, J = 7.6 Hz, 2H), 4.11 (s, 2H), 3.58 (s, 4H), 2.47 (s, 3H), 1.39 (d, J = 4.8 Hz, 3H), 1.25 (d, J = 4.7 Hz, 3H), 0.84 (s, 3H).

**Synthesis of 5-(azidomethyl)-2,2,5-trimethyl-1,3-dioxane (4):** Compound (3) (14.6 g, 46.4 mmol), NaN_3_ (12.1 g, 186 mmol), water (10 ml), and DMF (80 ml) were stirred at 110°C for 48 h under reflux. The mixture was poured into 150 ml water and extracted four times with Et_2_O (4 × 200 ml). The organic phase was dried over anhydrous MgSO_4,_ and the solvent was removed under reduced pressure. The residue was purified by column chromatography with silica gel (100 g) and ethyl acetate/n-hexane (1:4) to give 7.48 g of a colorless liquid with an 87% yield. ^1^H NMR (500 MHz, CDCl_3_) δ 3.58 (d, J = 2.8 Hz, 4H), 3.51 (s, 2H), 1.40 (d, J = 13.8 Hz, 6H), 0.81 (d, J = 1.1 Hz, 3H).

**Synthesis of 2-(azidomethyl)-2-methylpropane-1,3-diol (5):** Compound (4) (7.05 g, 40.3 mmol) was dissolved in 35 ml of methanol. 7.00 g of a Dowex, acid resin was added, and the reaction mixture was stirred for 12 h at 50°C. When the reaction was complete, the Dowex was filtered off in a vacuum filter under a low vacuum and carefully washed with methanol. The methanol was evaporated to give 5.41 g of white crystals with a 93% yield. ^1^H NMR (400 MHz, CDCl_3_) δ 3.73–3.58 (m, 4H), 3.56–3.43 (m, 2H), 2.19 (s, 2H), 0.89 (d, J = 2.0 Hz, 3H).

**Synthesis of A-MPA-4-AC (6):** 2,2,5-trimethyl-1,3-dioxane-5-carboxylic acid was prepared using similar method mentioned in compound (2) synthesis. 2,2,5-trimethyl-1,3-dioxane-5-carboxylic acid (6.48 g, 37.2 mmol), 1,1′-carbonyldiimidazole (CDI) (9.05 g, 55.8 mmol) were dissolved in 30 ml of ethyl acetate and it was stirred 1 h at 50°C. CsF (0.75 g, 4.93 mmol), Compound (5) (1.80 g, 12.4 mmol) were dissolved in 10 ml of ethyl acetate separately, and it was slowly added to the reaction mixture under nitrogen at 50°C. It was stirred for 12 h. When the reaction was complete 200 ml DI water was added and allowed to stir for 2 h at room temperature. Then it was extracted with 1 M HCl (200 ml × 3), 1 M NaHSO_4_ (200 ml × 3), 10% Na_2_CO_3_, saturated NaCl (200 ml), and it was dried under anhydrous MgSO_4_. Ethyl acetate was evaporated to give 5.22 g of colorless oil liquid with a 92% yield. ^1^H NMR (500 MHz, CDCl_3_) δ 4.21 (d, J = 11.7 Hz, 4H), 4.13–4.09 (m, 4H), 3.68 (d, J = 11.7 Hz, 4H), 3.42–3.39 (m, 2H), 1.43 (d, J = 32.2 Hz, 12H), 1.18 (d, J = 1.6 Hz, 6H), 1.08 (d, J = 1.5 Hz, 3H).

**Synthesis of A-MPA-4-OH (7):** Compound (6) (5.00 g, 10.9 mmol) was dissolved in 20 ml of methanol. 5.00 g of a Dowex, acid resin was added, and the reaction mixture was stirred for 12 h at 50°C. When the reaction was complete the Dowex, acid resin was filtered off in a vacuum filter under a low vacuum and carefully washed with methanol. The methanol was evaporated to give 3.96 g of colorless liquid with a 96% yield. ^1^H NMR (300 MHz, CDCl_3_) δ 4.09 (s, 4H), 3.88 (d, J = 11.2 Hz, 4H), 3.78–3.67 (m, 4H), 3.43 (s, 4H), 3.38 (s, 2H), 1.12–1.09 (m, 6H), 1.09–1.07 (m, 3H).

**Synthesis of A-MPA-4-ala (8):***N*-boc-alanine (3.70 g, 19.6 mmol), 1,1′-carbonyldiimidazole (CDI) (3.49 g, 21.5 mmol) were dissolved in 30 ml of dry ethyl acetate, and it was stirred for 1 h at 50°C. CsF (0.43 g, 2.81 mmol), compound (7) (1.23 g, 3.26 mmol) were dissolved in 5 ml of dry ethyl acetate and it was slowly added to the reaction mixture under nitrogen at 50°C. It was stirred for 12 h. When the reaction was complete 250 ml DI water was added and allowed to stir for 2 h at room temperature. Then it was extracted with 1 M HCl (200 ml × 3), 1 M NaHSO_4_ (200 ml × 3), 10% Na_2_CO_3_, saturated NaCl (200 ml), and it was dried under anhydrous MgSO_4_. Ethyl acetate was evaporated to give 3.15 g with a 91% yield. ^1^H NMR (400 MHz, CDCl_3_) δ 5.19 (s, 4H), 4.31–4.19 (m, 8H), 4.02 (d, *J* = 2.4 Hz, 4H), 3.38 (q, *J* = 6.2 Hz, 8H), 3.33 (s, 2H), 2.55 (t, *J* = 5.9 Hz, 8H), 1.27 (s, 6H), 1.02 (s, 3H).

### Synthesis Route of TRPZ-bisMPA

**Synthesis of 3,4-dichloro-1-(prop-2-yn-1-yl)-1H-pyrrole-2,5-dione (9)**: A total of 0.40 g of dichloromaleimide (2.40 mmol) and 2-ethylhexylbromide (0.48 ml, 2.9 mmol) and DMF (10 ml) in a 2-neck round bottom flask under nitrogen. The mixture was vigorously stirred at 140°C for 24 h. After that, the solution was quenched with 0.1 M HCl and extracted with diethyl ether. The organic layer was dried over Na_2_SO_4_ and concentrated under reduced pressure, and purification by silica gel column chromatography (30% DCM/Hexane) resulted in a cream-colored powder of *N*-(propargyl) dichloromaleimide (0.554 g, 90%). ^1^H NMR (400 MHz, CDCl_3_) δ 4.31–4.30(d), 2.23(s).

**Synthesis of TRPZ-Propagyl (10)**: A mixture of 2-aminothiophenol (0.386 g, 2.8 mmol) and *N*-(propargyl) dichloromaleimide (0.30 g, 1.4 mmol) was dissolved in 24 ml of acetic acid. The reaction mixture was refluxed under a nitrogen atmosphere for 24 h. After being cooled to room temperature, the mixture was quenched with brine and extracted with ethyl acetate. The organic layer was separated, dried over anhydrous Na_2_SO_4_, and concentrated under reduced pressure. The product was purified via flash column chromatography (30% chloroform/hexane) and obtained as orange solid (0.18 g, 50%). ^1^H NMR (400 MHz, chloroform-*d*) ^1^H NMR (400 MHz, CDCl_3_) δ 7.72 (d, J = 8.0 Hz, 2H), 7.34–7.26 (m, 4H), 7.18 (ddd, J = 8.3, 7.2, 1.4 Hz, 2H), 5.20 (s, 2H), 2.29 (t, J = 2.5 Hz, 1H). ^13^C NMR (75 MHz, CDCl_3_) δ 148.79, 136.97, 130.48, 128.26, 127.11, 126.57, 111.17, 77.23, 73.36, 33.08. ^1^H–^1^H correlation spectroscopy (COSY) spectrum is provided in the SI. HRMS (ESI/Q-TOF) m/z: [M + H]^+^ Calcd for C_19_H_11_N_3_S_2_ 345.0394; Found 345.0365 with an isotope pattern similar to the predicted pattern.

**Synthesis of TRPZ-bisMPA-amine-BOC (11):** This procedure was modified from previously reported procedures (Ihre et al., 1998). To the stirred solution of A-MPA-4-ala (0.60 g, 0.56 mmol), TRPZ-propagyl (0.29 g, 0.84 mmol) in 8 ml of DMF, CuBr (322 mg, 2.21 mmol) was added under nitrogen flushing. After complete addition, *N,N,N′,N″,N″*-pentamethyldiethylenetriamine (PMDETA) (388 mg, 2.30 mmol) was added and allowed to stir under nitrogen at 55°C for 48 h. The reaction mixture was precipitated to 200 ml diethyl ether. After settling, the diethyl ether layer was decanted, and the remaining product was air-dried. This precipitation, decanting, and the air-drying process was repeated twice more. The crude product was dissolved in 100 ml of DCM. It was extracted with 0.1 M EDTA solution. It was further purified using size exclusion chromatography (Sephadex LH-20) to give 0.59 g of TRPZ-bisMPA-amine-Boc with 74% yield. ^1^H NMR (400 MHz, DMSO-*d*_*6*_) δ 8.03 (s, 1H), 7.51 (d, *J* = 7.9 Hz, 2H), 7.44 (d, *J* = 8.0 Hz, 2H), 7.32 (t, *J* = 7.6 Hz, 2H), 7.22 (t, *J* = 7.6 Hz, 2H), 6.77 (s, 4H), 5.32 (s, 2H), 4.41 (s, 2H), 4.16 (d, *J* = 11.9 Hz, 8H), 3.94 (s, 4H), 3.12 (d, *J* = 6.4 Hz, 8H), 2.41 (t, *J* = 7.4 Hz, 8H), 1.34 (s, 36H), 1.17 (d, *J* = 20.4 Hz, 6H), 0.84 (s, 3H). HRMS (ESI/Q-TOF) m/z: [M + H]+ Calcd for C_66_H_90_N_10_O_20_S_2_ 1407.5852; Found 1407.5813 with an isotope pattern similar to the predicted pattern.

**Synthesis of TRPZ-bisMPA (12):** TRPZ-bisMPA-amine-Boc was dissolved in 5 ml of chloroform and 2 ml of trifluoroacetic acid (TFA) was slowly added and stirred for 45 min. The reaction mixture was air-dried and dissolved in 10 ml of chloroform. It was added dropwise to 500 ml of diethyl ether and stirred for 2 h. It was filtered, and the precipitation procedure was repeated three times. Finally, the resulting solid was put under the vacuum for 24 h to give 0.39 g of the pure product with 93% yield. The formation of the **TRPZ-bisMPA** was confirmed via the disappearance of BOC groups (1.34 ppm). ^1^H NMR (400 MHz, DMSO-*d*_*6*_) δ 8.07 (s, 1H), 7.53 (d, *J* = 7.8 Hz, 2H), 7.44 (d, *J* = 7.8 Hz, 2H), 7.33 (t, *J* = 7.7 Hz, 2H), 7.24 (t, *J* = 7.6 Hz, 2H), 5.33 (s, 2H), 4.42 (s, 2H), 4.22 (d, *J* = 11.5 Hz, 8H), 3.94 (s, 4H), 3.01 (q, *J* = 6.2 Hz, 8H), 2.64 (d, *J* = 7.1 Hz, 8H), 1.17 (s, 6H), 0.85 (s, 3H).

### Preparation and Characterization of Nanoparticles

For the nanoprecipitation method (Chandrasiri et al., 2020), 100 μL tetrahydrofuran (THF) was used as the organic solvent to dissolve 2 mg of **TRPZ-bisMPA** separate glass vial. The solution was added dropwise to a separate vial of Milli-Q water at pH 7.0 (2 ml) while gently stirring to obtain a 1 mg/ml final concentration. THF was allowed to evaporate under a stream of nitrogen. NP solutions were allowed to equilibrate for 12 h before further study.

**TRPZ-127 NPs** was prepared and modified according to the previously reported procedure (Wu et al., 2017). First, **TRPZ-PG** (2 mg) was dissolved under rapid sonication in THF (2 ml). Then, a THF solution (1 ml) containing **TRPZ-PG** (1 mg/ml) and Pluronic F-127 (5 mg/ml) was used to prepare **TRPZ-127 NPs** by rapidly injecting the solution into deionized water (1 ml) under continuous sonication using a bath sonicator. After sonication for an additional 1 min, THF was evaporated under a nitrogen atmosphere.

Aggregate sizes and ζ-potentials measurements were carried out on a Malvern Instrument Zetasizer Nano ZS using a He–Ne laser with a 633 nm wavelength, a detector angle of 173^o^ at 25°C. The size measurements were performed in triplicate for each sample at 0.5 mg/ml concentration to ensure consistency. The morphological study of the aggregates formed from the NPs was carried out by TEM using a JEOL 1230 TEM operated at 100 kV to collect the TEM images using a Gatan Orius 831 bottom mounted CCD camera.

### Absorption and Photoluminescence Assessment

The absorption measurements were done on a Varian Cary-5000 spectrometer (Dorval, QC, Canada). While the fluorescence studies were performed on Horiba Quantamaster fluorimeter with a xenon lamp and PMT detector using glass cuvettes. Fluorescence quantum yields were measured with samples of low sample concentration (10^–5^ M) and excited close to their maximum absorption. The spectroscopic energy gap ($E_{g}^{opt}$) was calculated from the onset of absorbance (Li et al., 2012).

### Cell Viability, Treatment and Imaging

Human embryonic kidney (HEK293) cells were used for the assay. HEK cells were grown under standard conditions (37°C, 5% CO_2_, DMEM media with 10% FBS). Nanoparticles were added to tissue culture media and allowed 24 h incubation period in cytotoxicity studies. Cytotoxicity of the nanoparticles was evaluated with a CyQUANT LDH Cytotoxicity Assay Kit (Invitrogen) using a microplate reader (BioTek Synergy H1). Following manufacturer protocols, both negative and positive controls are used in the assay. Experimental values are transformed based on two values: zero-cytotoxicity value (background) and 100% cytotoxicity value (cells treated with lysis buffer based on manufacturer protocol). Each experiment is represented by relative values based on the control values. Imaging of particle distribution in cells was done 30 min after the addition of 10 μg/ml NPs to culture media. TRPZ fluorescence in HEK cells was observed with a Leica Stellaris STED confocal microscope using both conventional and STED modes.

## Results and Discussion

### Design and Synthesis

TRPZ, first discovered by Dimroth and Reicheneder (1969), possesses a simple yet fascinating molecular structure. Its pi-framework and redox properties have made it beneficial in the field of organic electronics. TRPZ, being planar in structure, can undergo efficient crystalline packing via intermolecular sulfur–sulfur interactions and hydrogen bonding between pyrrole hydrogens and thiazine nitrogens (Hong et al., 2008; Hong et al., 2009). The molecule has multiple reactive sites which can be functionalized to increase its solubility and be further developed using electron-withdrawing and electron-donating groups to afford donor-acceptor oligomers with exceptional optical properties.

Based on its unique properties, we aimed to expand the application of this unique emissive building block towards bioimaging. However, due to its hydrophobic nature, we sought to modify TRPZ via N-substitution of the central pyrrole to produce a self-assembling amphiphile. To induce amphiphilicity and promote self-assembly, bisMPA was selected as the dendritic segment. A hydrophilic moiety, its synthetic accessibility allows esterification under mild reaction conditions and provides a level of biodegradability and biocompatibility (Feliu et al., 2012). Additionally, it was capped with alanine to afford a cationic surface to increase colloidal stability, cellular uptake and better its water solubility (Gessner et al., 2002; Lipfert et al., 2014; Dragoman et al., 2017). This amphiphilic property is expected to make TRPZ capable of forming NPs independently, **TRPZ-bisMPA NPs**.

The resulting NP morphology is anticipated to be that of a micelle structure in which the dendron extends towards the hydrophilic water surface protecting the hydrophobic TRPZ portion within the core. The positively charged terminal ends of the amphiphile would effectively stabilize the NPs via electrostatic interactions.

In parallel, we compared NPs formed from **TRPZ-bisMPA** with conventional nanostructures in which a surfactant polymer is employed to encapsulate hydrophobic dyes. We encapsulated **TRPZ-PG** into a water-soluble polymer surfactant (Pluronic F-127), **TRPZ-127 NPs**. Although frequently employed, the encapsulation strategy has significant drawbacks (e.g., poor loading) that limit its application. Often the nature of the molecule being encapsulated has varying physiochemical properties that contrast to the solubilizing block of the amphiphilic polymer being employed. This, of course, leads to lower encapsulation efficiencies and can destabilize the resulting nanostructure (Jeong et al., 2020). By assessing the two NP systems, **TRPZ-bisMPA NPs** and **TRPZ-127 NPs**, we aim to explore the former as a versatile molecular bioimaging probe suitable for numerous biomedical applications.

**SCHEME 1:**
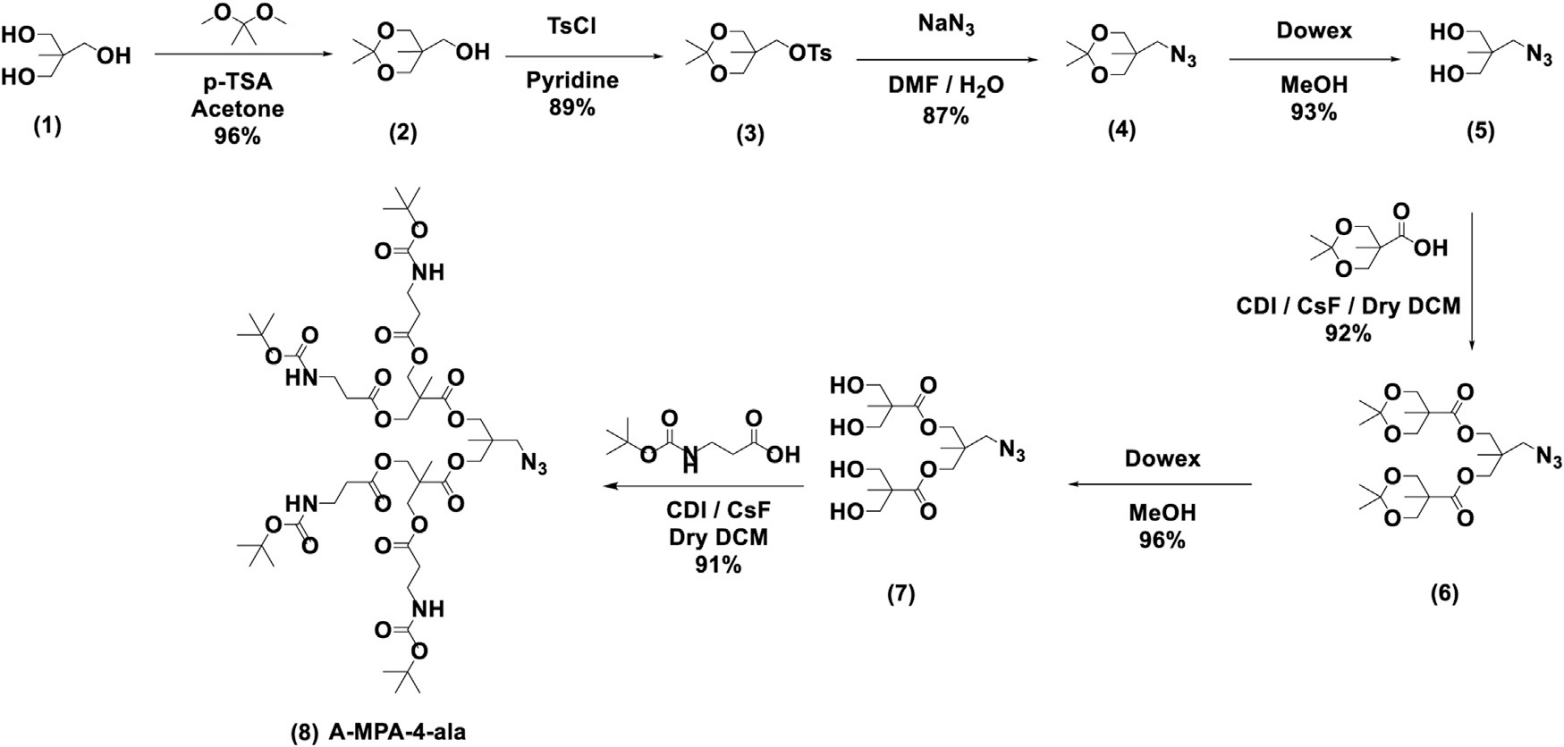
Synthesis of dendron segment, A-MPA-4-ala; the synthesis was modified accordingly using previously reported procedures (Yaddehige et al., 2020).

The synthesis of the dendron segment, A-MPA-4-ala (8), is shown in Scheme 1, which consists of a seven-step synthetic route. In the first step, the 1,3-diol moiety of bisMPA (1) was protected to afford compound 2 by reaction of bisMPA with 2,2-dimethoxypropane and a catalytic amount of p-toluenesulfonic acid (TsOH) in dry acetone. In order to make the hydroxyl group a better leaving group, compound 2 was reacted with p-toluenesulfonyl chloride (TsCl) in pyridine to obtain compound 3. The next step, the azido group, was substituted using sodium azide (NaN_3_) and DMF to obtain compound 4. The following step was carried out to deprotect dimethoxy moiety using an anion exchange resin-Dowex to obtain compound 5. Before proceeding to the next step, 2,2,5-trimethyl-1,3-dioxane-5-carboxylic acid was synthesized using a similar method to compound 2. Then, 2,2,5-trimethyl-1,3-dioxane-5-carboxylic acid was employed in a Malkoch esterification (García-Gallego et al., 2015) with compound 5, using 1,1′-carbonyldiimidazole (CDI) to activate the carbonyl group and cesium fluoride (CsF) as a catalyst to obtain compound 6. Dowex deprotection was done in the next step to obtain compound 7. The final step was esterification between beta-alanine and compound 7 to obtain A-MPA-4-ala.

**SCHEME 2:**
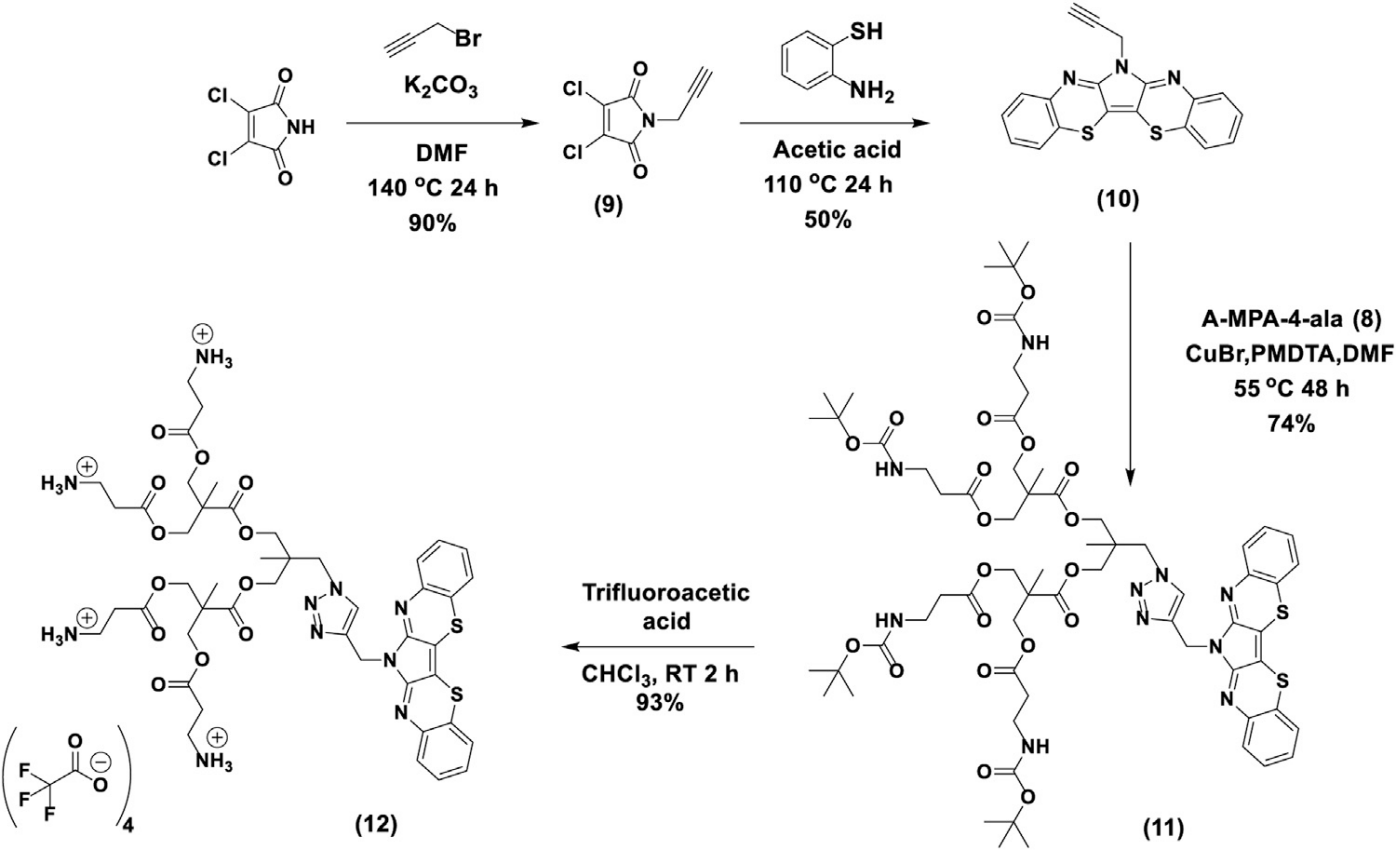
Synthesis route for TRPZ-bisMPA.

The synthesis of **TRPZ-bisMPA** (12) consists of four synthetic steps (Scheme 2). First, dichloromaleimide was propargylated at the nitrogen position to obtain compound 9. **TRPZ-PG** was synthesized *via* a condensation method between 2-aminothiophenol and *N*-(propargyl) dichloromaleimide (compound 10). Then **TRPZ-PG** (10) and A-MPA-4-ala (8) were reacted using click-chemistry to obtain TRPZ-bisMPA-amine-Boc (compound 11). Finally, acid was used for deprotection of the tert-butyl carbamates (BOC) protected amine groups to afford a cationic terminated **TRPZ-bisMPA** amphiphile (compound 12).

#### Self-Assembly and Nanoparticle Formation

**FIGURE 2:**
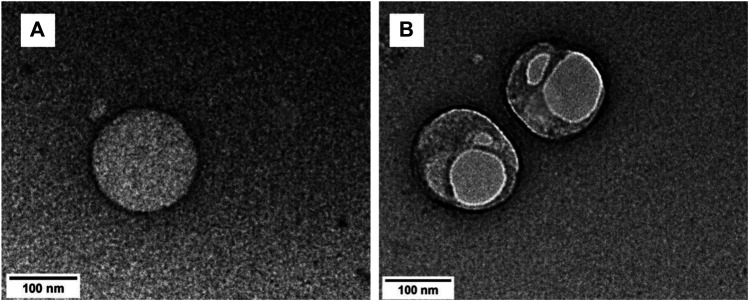
TEM image of a TRPZ-bisMPA NPs **(A)** and TEM image of a TRPZ-127 NPs **(B)** in Milli-Q water from nanoprecipitation method. Additional images are provided in the SI.

The particle formation for **TRPZ-bisMPA** was done by nanoprecipitation. The hydrodynamic diameter was analyzed via dynamic light scattering (DLS) (Supplementary Figure S8A). Supplementary Table S1 summarizes the sizes and surface charges for the NPs formed. Nanoprecipitation affords NPs with a spherical morphology possessing a hydrodynamic diameter of 129.9 nm (±20). The polydispersity (PDI) for **TRPZ-bisMPA NPs** indicated high uniformity with values around 0.2. Figure 2A shows transmission electron microscopy (TEM) images for **TRPZ-bisMPA NPs**. TEM images support DLS data and provide direct evidence of NP formation with a diameter of 155.1 nm (±16).

For comparison, **TRPZ-PG** was encapsulated with Pluronic F-127 (Mw = 12.6 kDa). It is not readily soluble in water unless incorporated within a water-soluble surfactant. Pluronic F-127 is known to self-assembly independently with sizes that are much smaller than those observed in this study (<50 nm) (Domínguez-Delgado et al., 2016). The DLS data indicates a larger NPs assembly than that of **TRPZ-bisMPA NPs. TRPZ-127 NPs** exhibit a hydrodynamic diameter of 323.5 nm (±97 nm) (Supplementary Figure S8B) and PDI of 0.19. TEM images of **TRPZ-127 NPs** show spherical assemblies with average diameters of 181.5 nm (±66) (Figure 2B). The encapsulation efficiency (EE%) of 38.5% and dye loading efficiency (DL%) of 7.1% are tabulated in the SI.

Surface properties of the NPs *via* ζ-potential values aid in supporting the sizes obtained from DLS and TEM. The ζ-potential, which depends on the surface charge, is essential for the stability of NPs in suspension. Additionally, it is a critical factor in regards to the function and toxicity of NPs specifically for biological application. TRPZ-bisMPA NPs exhibit ζ-potentials of 17.2 mV. The positive charge results from the outer surface consisting of cationic amines. In contrast, TRPZ-127 NPs show -0.5 mV of average surface charge, which is close to neutral due to the lack of charge on the polymer surfactant used. The positive surface charge on TRPZ-bisMPA NPs corresponds to small and more stable aggregates (Kumar and Dixit, 2017).

#### Photophysical Properties

**FIGURE 3:**
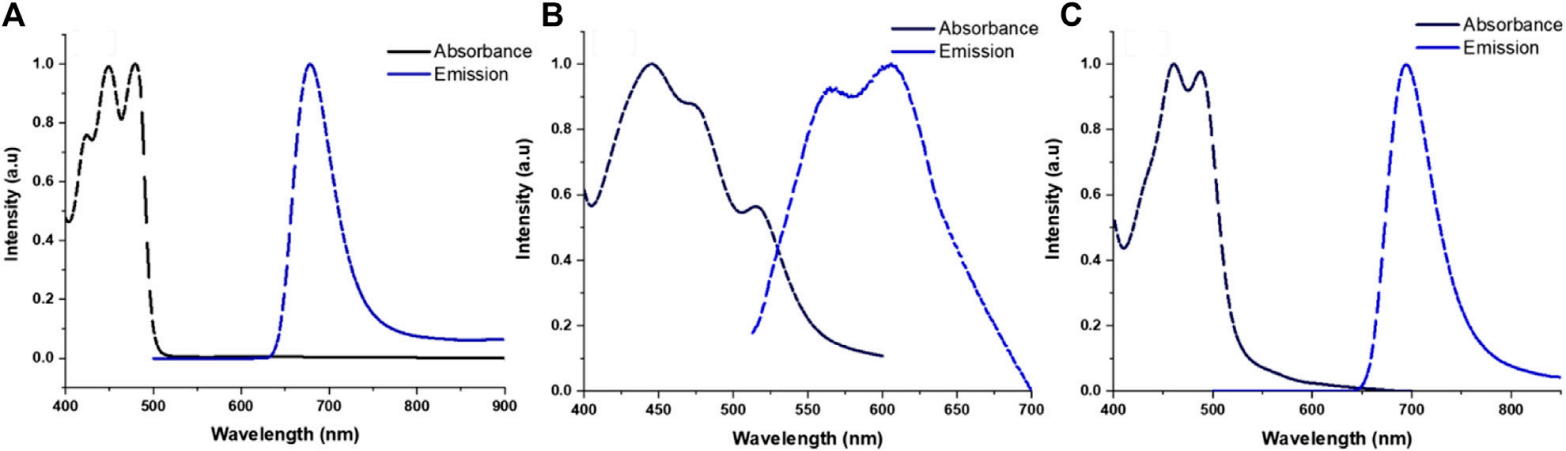
Normalized absorbance and emission profiles for TRPZ-PG **(A)** in THF, TRPZ-127 NPs **(B)** and TRPZ-bisMPA NPs **(C)** in Milli-Q water.

The normalized absorption and emission spectra of TRPZ and its NPs are shown in Figure 3 with additional photophysical data tabulated in Table 1. In a comparison of **TRPZ-PG** fluorophore with the two NP suspensions, **TRPZ-PG** shows two major absorbance peaks at 447 and 478 nm in THF (Figure 3A). The band at 447 nm can be assigned to the pi to pi* electronic transition of the phenyl rings. Bands at 478 nm are assigned to the n to pi* transitions. The calculated optical band gap of the **TRPZ-PG** is 2.46 eV (onset: 504 nm). The emission maximum of the fluorophore is at 678 nm with a Stokes shift of 0.76 eV (200 nm). Note that the propargyl group does not contribute to the observed photophysical properties via pi orbital overlap.

**TABLE 1:** Optical properties of the TR-PZ systems dye in THF^a^ and NPs in water^b^.

TR-PZ systems	λ_abs_^max^ (nm)	E_g_^opt^ (eV)	λ_ems_^max^ (nm)	Stokes shift (nm, eV)	*Φ* (%)	τ (ns)
TRPZ-PG^a^	447, 478	2.46	678	200 (0.76)	79	5.3
TRPZ-127 NPs^b^	452, 480, 515	2.48	563, 606	126 (0.54)	<0.5	t_1_ = 0.8 (66.9%),t_2_ = 5.7 (33.1%)
				83 (0.38)		
TRPZ-bisMPA NPs^b^	460, 494	2.52	695	201 (0.72)	49	6.3

For the NPs, noticeable broadening and red-shifting (2–16 nm) in the spectra suggest enhanced intermolecular interactions due to aggregation in the NP core. **TRPZ-127 NPs** display three absorbance peaks at 452 nm, 480 nm, and 515 nm in water, as shown in normalized absorbance spectra (Figure 3B). Compared to both **TRPZ-PG** and **TRPZ-bisMPA NPs**, a peak shoulder at 515 nm is observed, presumably due to a differing aggregation pattern within the particle. **TRPZ-127 NPs** possess a weak emission and is blue-shifted towards 563 and 606 nm relative to **TRPZ-PG**.

Interestingly there is a contrast in optical properties between the amphiphile and encapsulated **TRPZ**. **TRPZ-bisMPA NPs** show absorbance peaks at 460 and 494 nm in water (Figure 3C). Upon excitation, **TRPZ-bisMPA NPs** exhibit a strong neon green fluorescence with an emission band centered at 695 nm and a Stokes shift of 0.72 eV (201 nm). The optical profile for **TRPZ-bisMPA NPs** resembles that of “free” **TRPZ-PG** in THF, with a 20 nm red shift for the latter. This behavior is presumably due to an aggregation behavior that is reminiscent of a monomeric form (e.g., “free” or non-aggregated **TRPZ-PG** form) of the dye with extended electron delocalization. The weaker emission of **TRPZ127 NPs** relative to **TRPZ-bisMPA NPs** is supported by a negative solvatochromism observed for **TRPZ-PG** (Supplementary Figure S13). These results suggest that **TRPZ-PG** possesses a nonpolar excited state and can form high-energy aggregates (e.g., H-aggregates) that are not dominant with the amphiphilic derivative.

The Stokes shifts observed for the molecules and NP suspension are quite significant. Large Stokes shifts are important when overcoming spectral overlapping to retain good signal-to-noise ratios for bioimaging applications. Additionally, a large Stokes shift can be indicative of a number of photophysical causes such as intramolecular charge transfer (ICT), (Yang et al., 2013), low reorganization energy, (Chen et al., 2019), and exciplex formation (Horváth et al., 2015). Among the aforementioned reasons, the Stokes shifts observed for TRPZ and its NPs are presumably due to the emission facilitated by the proaromatic pyrrole trapped in the quinoidal structure (Wu et al., 2010; Brogdon et al., 2016). Computational calculations for **TRPZ-bisMPA** support this notion where HOMO orbitals showed proaromatic molecular orbitals (MOs) and an aromatic excited state (LUMO) predominately at the pyrrole ring (Supplementary Figure S10). Electron localization function (ELF) and localized-orbital locator (LOL) analysis and profiles (Supplementary Figure S11) were employed to further support our hypothesis regarding proaromaticity. ELF has been widely used to evaluate electron localization while LOL offers insight on localized bonding features (Schmider and Becke, 2000; Tsirelson and Stash, 2002). Using the ELF-pi analysis, the aromatic rings display high electron delocalization where they are clearly separated from those with localized bonding. As expected, the ELF-pi values and analysis of bond orbitals confirm aromaticity. Additionally, localization functions show aromaticity following excitation which is a signature of proaromaticity (Cocq et al., 2015).

Along with a large Stokes shift, **TRPZ-bisMPA NPs** possess a high quantum yield (*Φ*) in water, *Φ* = 49%. Typically, the *Φ* for conventional bioimaging dyes are much lower in water due to unfavorable solvent-solute interactions, which induce non-radiative pathways and quench emission (Lakowicz, 2006; Hong et al., 2016). In this case, self-assembly of the amphiphile reduces dye-water interactions and aids to maintain the optical properties of a monomeric form of the dye (e.g., **TRPZ-PG**) (Zhegalova et al., 2014). **TRPZ-127 NPs** show weaker emission and lower *Φ* of 0.5% in water, most likely due to a variation in the aggregation patterns of TRPZ and the formation of H-aggregates inside the NP. **TRPZ-PG** fluorophore displays a decent *Φ* in organic solvents (*Φ* = 79% in THF). However, due to its poor solubility, comparison in aqueous media was not achievable.

Experimental lifetime plots are shown in SI (Supplementary Figures S14–S16). Fluorescent lifetime (τ) expresses the time allocated by a fluorophore in the excited state before relaxing to the ground state. Additionally, fluorescence lifetime measurements are highly sensitive to the surrounding environment (Boreham et al., 2016). **TRPZ-PG** in THF shows a signal exponential component of 5.3 ns and **TRPZ-bisMPA NPs** exhibits a lifetime of 6.3 ns. In contrast, **TRPZ-127 NPs** shows a dual exponential lifetime: t_1_ of 0.8 ns and t_2_ of 5.7 ns. The two exponentials are due to both the aggregated and monomeric (e.g., non-aggregated) form being present inside the NP. When compared with **TRPZ-PG** and the absence of a lower (t_1_) value, the lifetime of 0.8 ns can be assigned to high-energy aggregates, which in this case is the dominant form (66.9%).

### Cellular Viability, Uptake, and Imaging

**FIGURE 4:**
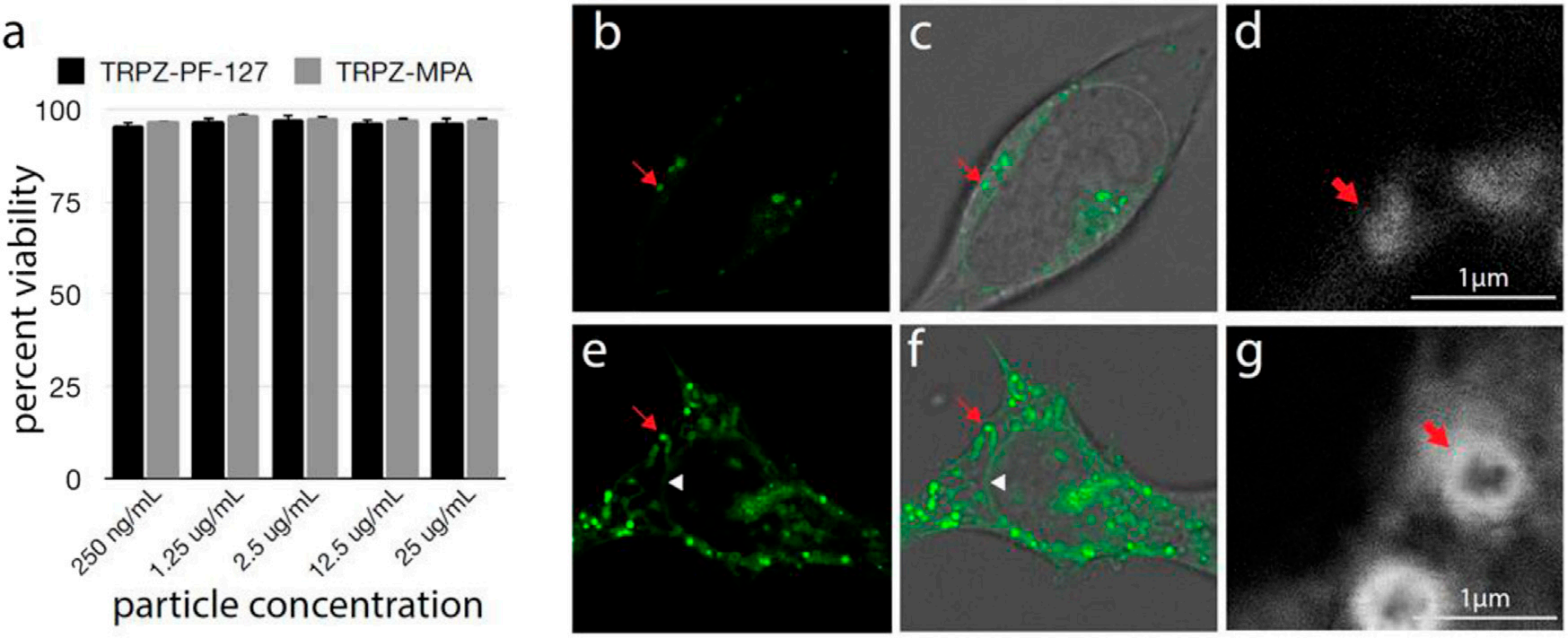
Cell viability and uptake of TRPZ nanoparticles **(A)** Percent cell viability after treatment with TRPZ-bisMPA NPs and TRPZ-127 NPs. Concentrations tested listed below. No significant difference was seen between any condition as determined by Tukey ANOVA **(B–G)**. Fluorescence microscopy of TRPZ-bisMPA NPs **(B–D)** and TRPZ-127 NPs **(E–G)** localization after cell loading. Images were acquired by conventional confocal microscopy **(B,E)** or STED **(D,G)**. Brightfield images were overlaid with fluorescence imaging **(C,F)**. Red arrow indicates a labeled lysosome. White triangle in TRPZ-127 NPs images shows nuclear membrane. Enlargement located in the SI.

The impact of the NPs on cell viability was tested by LDH assay (Figure 4A). Even at very high concentrations, a negligible effect was seen on cell viability. Analysis by Tukey ANOVA found no significant difference between conditions. *In vivo* testing will be needed to further assess the effect of the NPs on physiology; however, these results suggest our NPs will be well-tolerated in biological environments.

Confocal microscopy found that both **TRPZ-bisMPA NPs** and **TRPZ-127 NPs** accumulate most significantly in endosomes (Figures 4B–G). The cationic surface charge of **TRPZ-bisMPA NPs** leads to apparent cellular uptake via electrostatic interactions with anionic cell membranes. Similar results have been observed with other dendrimer-based NPs modified with anime groups (Morris et al., 2017; Ray et al., 2018; Ingle et al., 2020). Likely owing to the polar groups on the MPA dendron, TRPZ fluorescence is observed throughout the interior of the lysosome (Figure 4D).

**TRPZ-127 NPs** show not only accumulation in the lysosome but also labeling of various cellular membranes, including nuclear membranes (Figures 4E,F). Staining of the nuclear membrane indicates that TRPZ fluorescence has spread throughout the endomembrane system, trafficking all the way to the endoplasmic reticulum (ER). Stimulated emission depletion microscopy (STED) images of **TRPZ-127 NPs** and **TRPZ-bisMPA NPs** aid in comparing cellular uptake where fluorescence labeling is localized to the membrane of the organelle and not the interior, as observed with **TRPZ-bisMPA NPs** (Figures 4D,G; Supplementary Figure S17). It appears that **TRPZ-127** is much more aggressively loading into cells; however, this is not the case. We speculate that the encapsulated dye may have escaped its NPs and, due to its hydrophobic nature, became embedded into membrane interiors. The unloaded dye being hydrophobic actively segregates into cells while **TRPZ-bisMPA NPs** remain intact and localized in the lysosome.

## Conclusion

In summary, a comparative analysis of two NP systems was conducted. Evaluation via spectroscopic, light scattering and microscopic techniques confirm colloidal stability and provided insight on photophysical properties of the molecular scaffold as a potential bioimaging agent. Cell viability studies indicate low cytotoxicity of the materials and their suitability for biological application. However, cellular distribution of TRPZ originating from the two sets of NPs suggests a fundamentally different interaction with cells. Such results suggest that a variation in formulation could be used for different fundamental applications. Overall the approach described here opens up avenues towards developing fluorescent NPs from a simple yet appealing scaffold to afford materials with tuneable properties for bioimaging applications.

## Data Availability

The original contributions presented in the study are included in the article/Supplementary Material, further inquiries can be directed to the corresponding author.
