# Supplementary material for: Heteroacene-Based Amphiphile as a Molecular Scaffold for Bioimaging Probes
Source: Front Chem. 2021 Aug 18;9:729125. doi: 10.3389/fchem.2021.729125 (PMC8416430; doi:10.3389/fchem.2021.729125)
Supplement: Supplementary file 1 [file DataSheet1.PDF]

Supporting Information for "Heteroacene-based amphiphile as a molecular scaffold for bioimaging probes"

Tharindu A. Ranathunge[1], Mahesh Loku Yaddehige[1], Jordan H. Varma[1], Cameron Smith[1], Jay Nguyen[2], Iyanuoluwani Owolabi[2], Wojciech Kolodziejczyk[3], Nathan I. Hammer[1], Glake Hill[3], Alex Flynt[2], Davita L. Watkins[1*]

[1] Department of Chemistry and Biochemistry, University of Mississippi, University, Mississippi 38677, USA

[2]Cellular and Molecular Biology, University of Southern Mississippi, Hattiesburg, Mississippi 39406, USA

[3]Interdisciplinary Center for Nanotoxicity, Department of Chemistry, Physics and Atmospheric Sciences, Jackson State University, Jackson, Mississippi 39217, USA

*To whom correspondence should be addressed: dwatkins@olemiss.edu;

Table of Contents Pages

General Summary of Synthesis S2
Synthesis Details and NMR Spectra S2
Nanoparticle Characterization S10
Computational Data S12
Photophysical Characterization and Fluorescence Lifetime S16
Cellular Imaging S18
References S18

General Summary: Reagents and solvents were purchased and used without further purification unless otherwise specified: 3-hydroxy-2-(hydroxymethyl)-2-methylpropanoic acid (98%, Alfa Aesar), 2,2-dimethoxypropane (98%, Alfa Aesar), sodium azide (99%, VWR), 2-(hydroxymethyl)-2-methylpropane-1,3-diol (98%, Alfa Aesar). All synthetic procedures were performed under nitrogen atmosphere using standard Schlenk line techniques. Anhydrous solvents were obtained with a Glass Contour (Irvine, CA, USA) solvent purification system. Thin-layer chromatography was performed on SiO_2-60 F254 aluminum plates with visualization by ultraviolet (UV) light. Flash column chromatography was performed using Purasil SiO_2-60, 230−400 mesh from Whatman. Nuclear magnetic resonance (^{1}H NMR and ^{13}C NMR) spectra were performed on a Bruker Avance spectrometer (Bruker, Germany), operating at 500 Mhz, 400 MHz and 300 MHz with DMSO-d_6(δH = 2.50 ppm), MeOD (δH = 2.50 ppm), and $CDCl_3$ (δH =7.26 ppm) as NMR solvents and tetramethylsilane (TMS) as an internal standard. Data are reported as follows: chemical shift, multiplicity (s = singlet, br s = broad singlet, d = doublet, t = triplet, q = quartet, m = multiplet), coupling constants (Hz), and integration.

Synthesis Details and NMR Spectra

Synthetic protocol adopted from previously reported procedures.(Dimroth and Reicheneder, 1969; Yaddehige et al., 2020) Herein we report the NMR analysis of 9 to 12 synthetic steps.

NMR Analysis

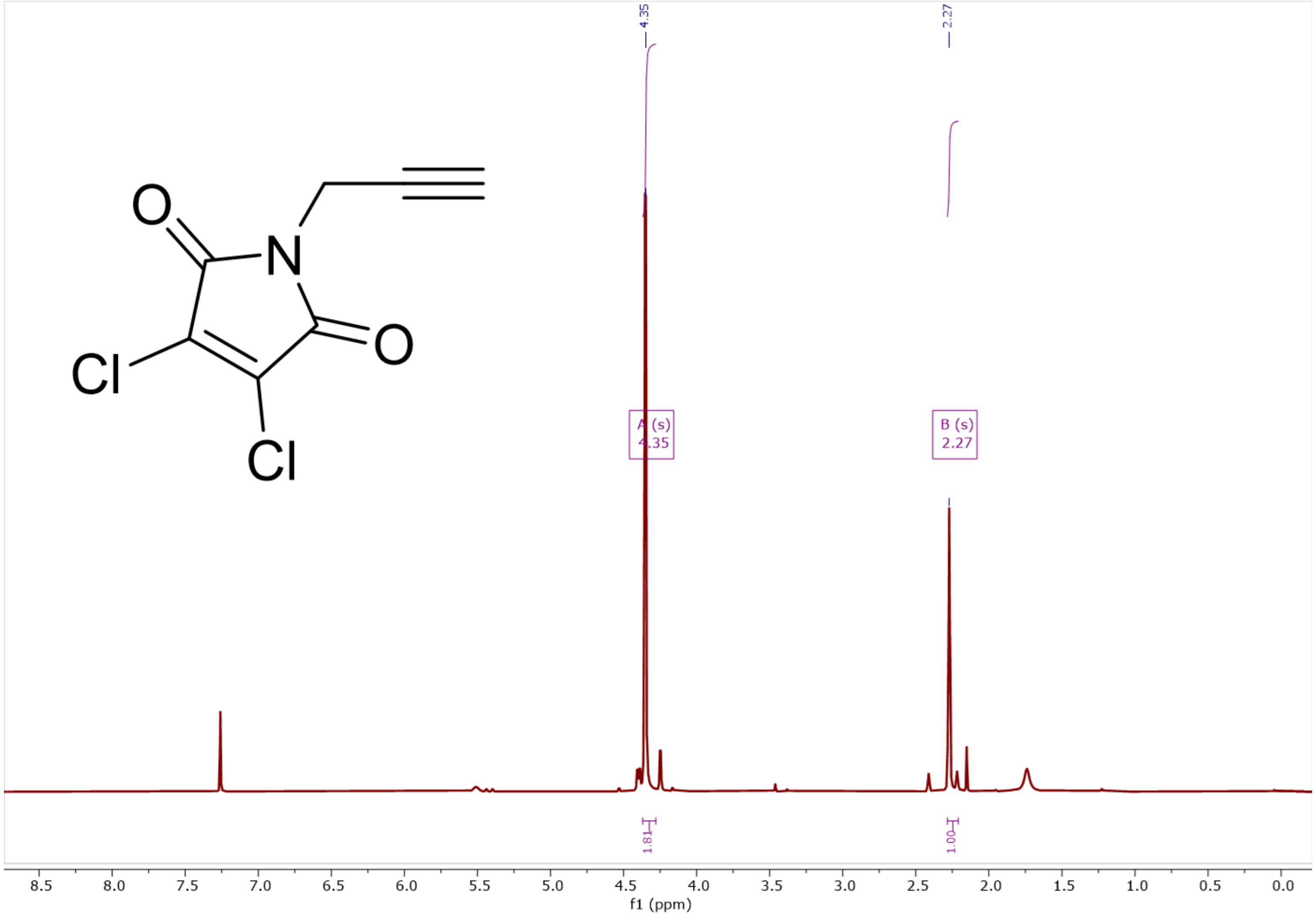

Figure S1. ^{1}H NMR spectrum of **TRPZ-PG** ($CDCl_3$, 400 MHz).

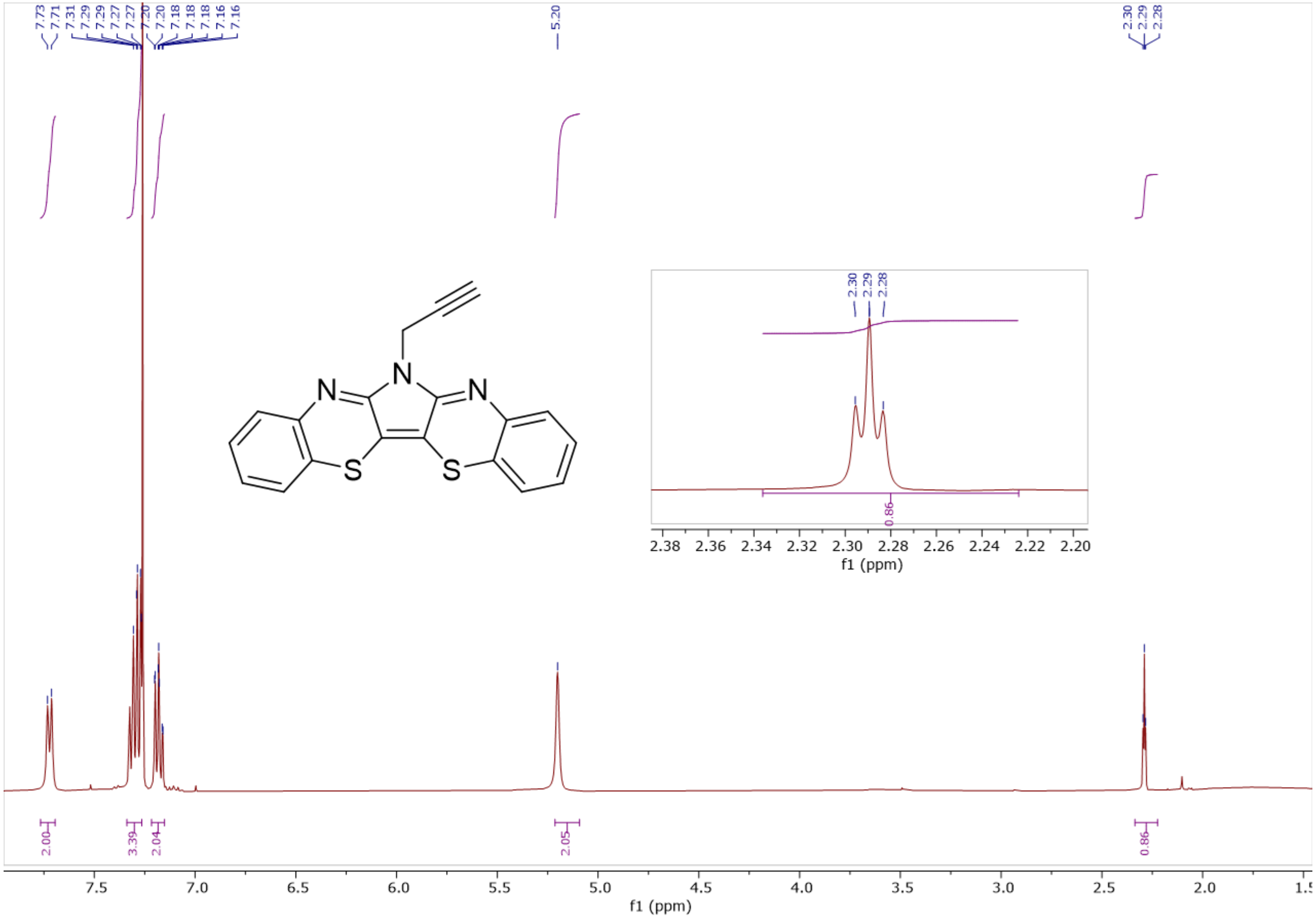

Figure S2. ^{1}H NMR spectrum of **TRPZ-PG** ($CDCl_3$, 400 MHz).

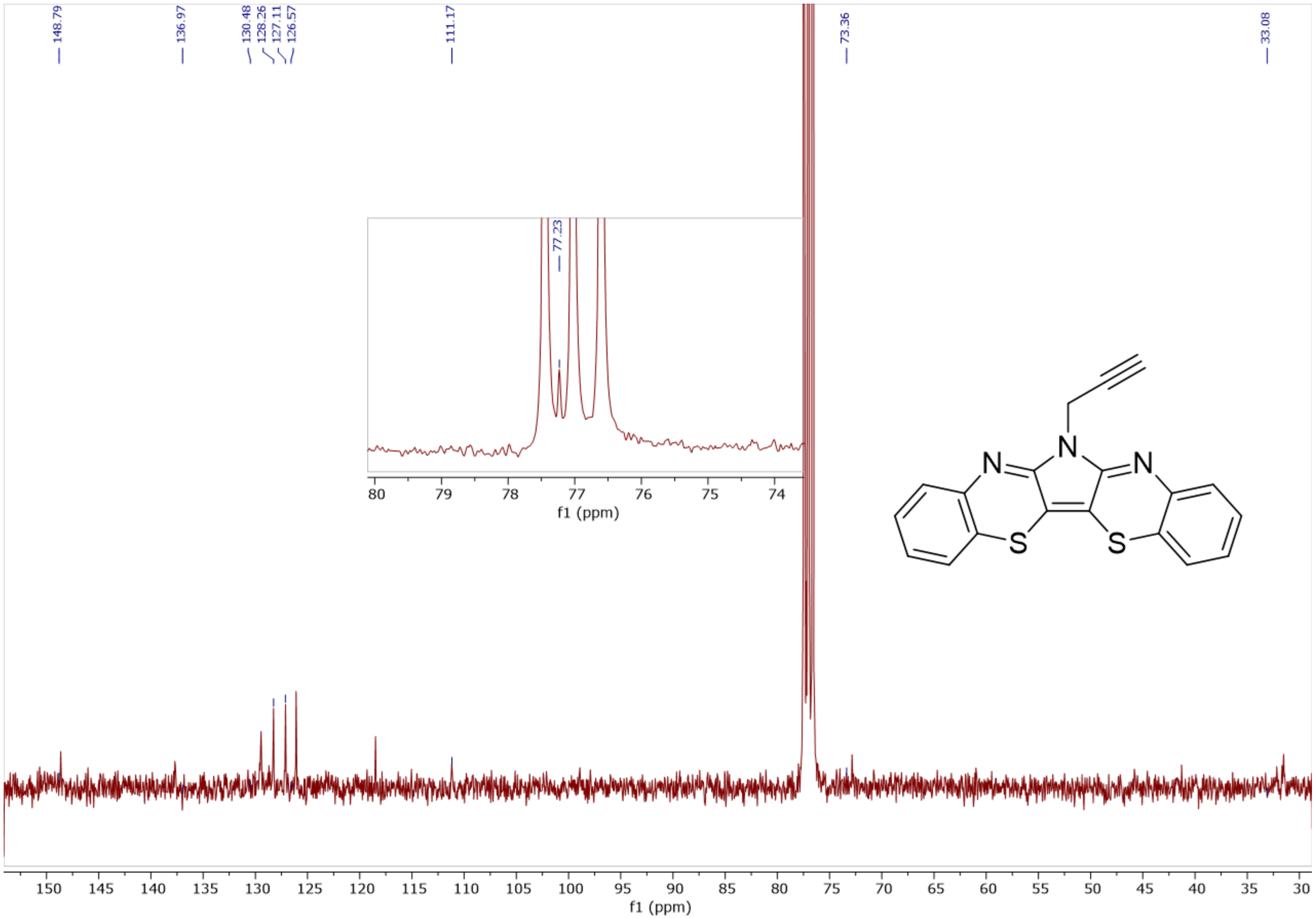

Figure S3. ^{13}C NMR spectrum of **TRPZ-PG** ($CDCl_3$, 75 MHz).

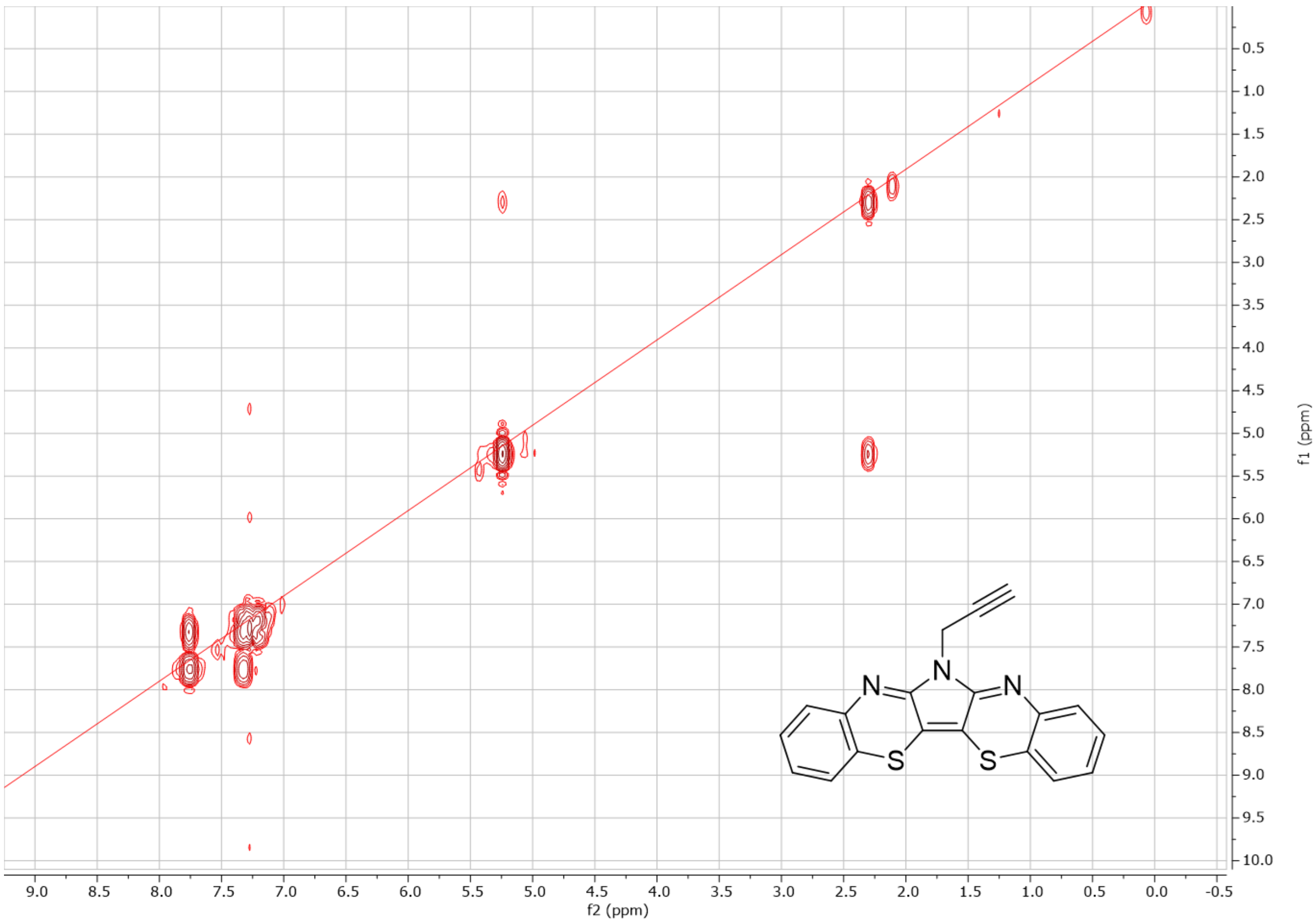

Figure S4. ^1H-^1H COSY NMR spectrum of **TRPZ-PG** ($CDCl_3$, 400 MHz).

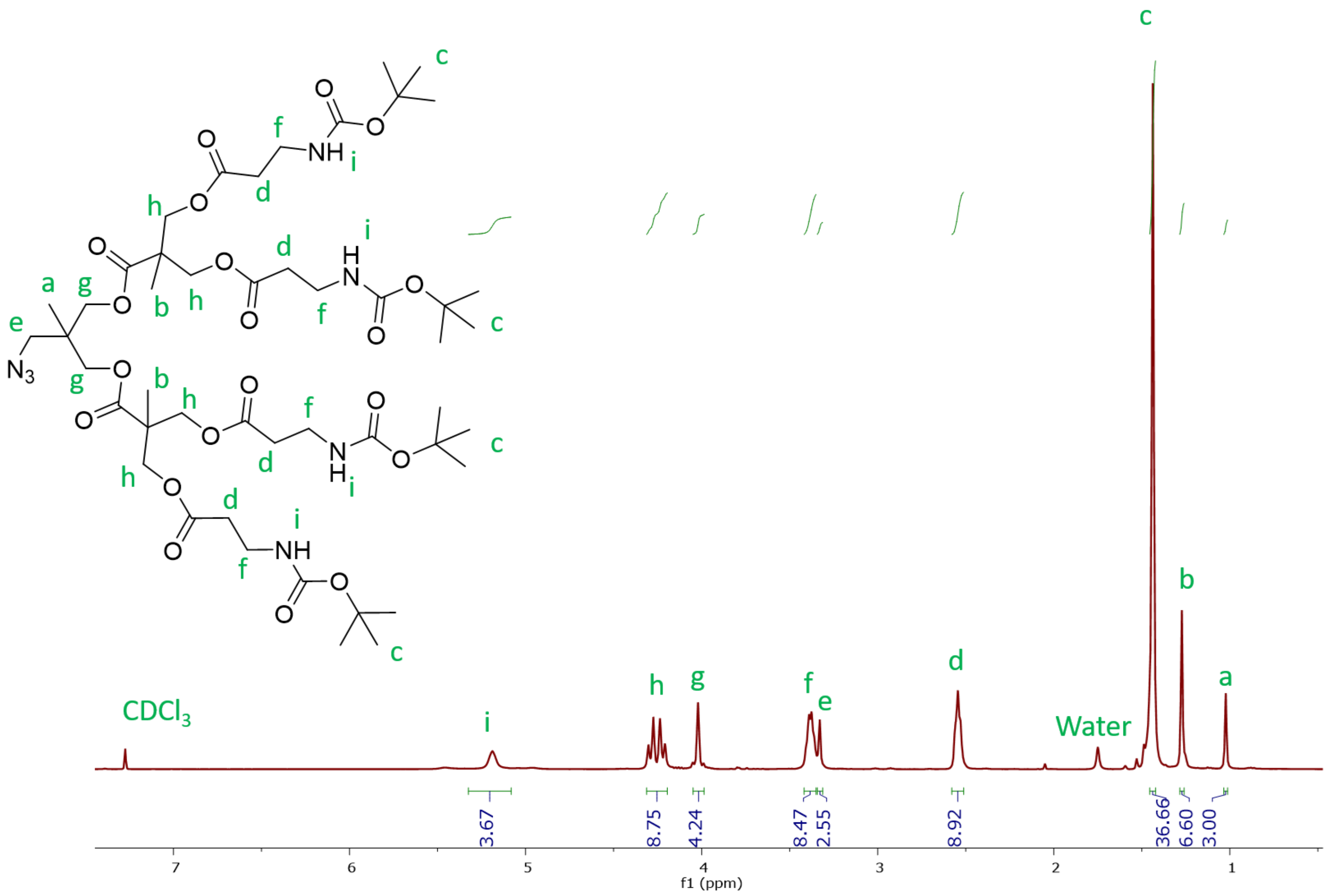

Figure S5. ^{1}H NMR spectrum of **A-MPA-4-ala** ($CDCl_3$, 400 MHz).

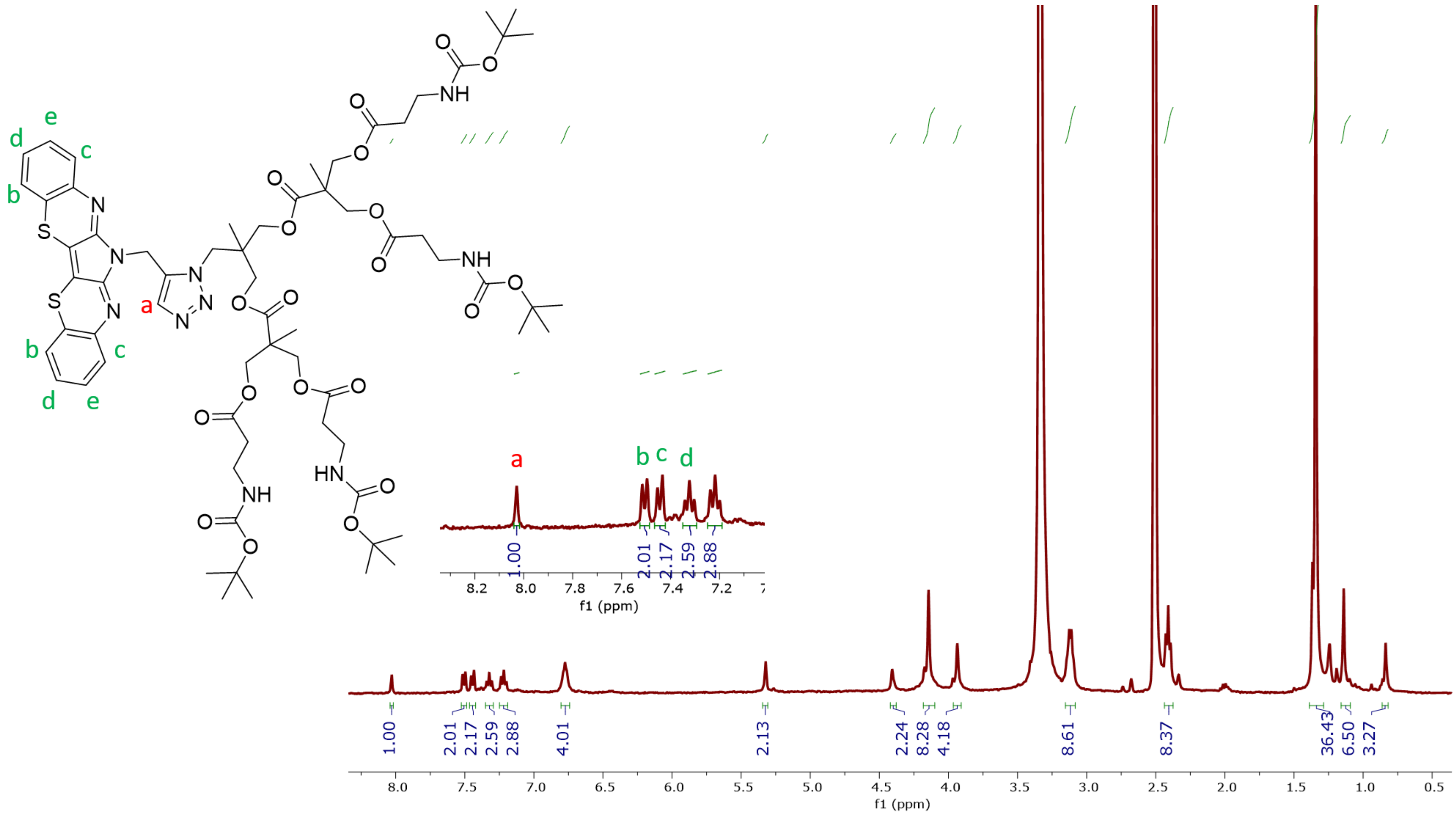

Figure S6. ^{1}H NMR spectrum of **TRPZ-bisMPA-amine-BOC** (DMSO-d_6, 400 MHz).

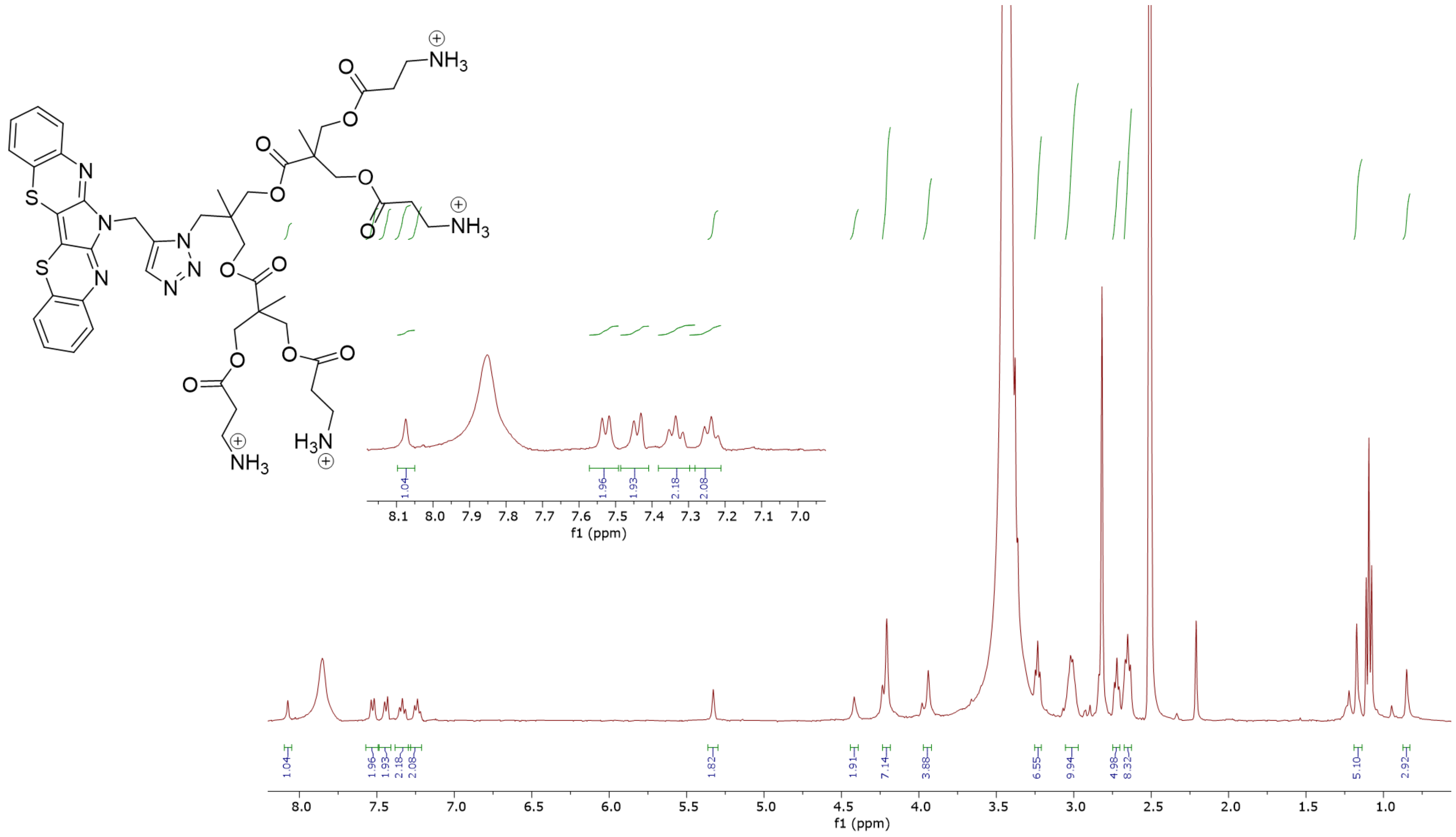

Figure S7. ^{1}H NMR spectrum of **TRPZ-bisMPA** (DMSO-d_6, 400 MHz) (Solvent impurity peaks are water, acetone diethyl ether).

TRPZ-PG encapsulation Study

The encapsulation efficiency (EE%) and dye loading efficiency (DL%) were determined for each molecular species. TRPZ-127 NPs were prepared and modified according to a previously reported procedure.(Wu et al., 2017) First, TRPZ-PG (2 mg) was dissolved under rapid sonication in THF (2 mL). Then, a THF solution (1 mL) containing TRPZ-PG (1 mg/mL) and Pluronic F-127 (5 mg/mL) was used to prepare TRPZ-127 NPs by rapidly injecting the solution into deionized water (1 mL) under continuous sonication using a bath sonicator. After sonication for an additional 1 min, THF was evaporated under a nitrogen atmosphere. Formulations were allowed to equilibrate for 12 h, and the unloaded dye was filtered out using a 0.45 µm syringe filter. Then, water was removed by freeze-drying, and the dye-loaded nanoparticles were redissolved in THF (5 mL).

The dye concentration was estimated by using a Cary 6000 UV−visible spectrophotometer at 480 nm based on the standard calibration curve obtained from free TRPZ-PG in THF. DL% and EE% were calculated using the below mentioned formula.

Encapsulation Efficiency (EE)

$$EE = \frac{m_{ed}}{m_{fd}} x100\%$$

m_{ed} –mass of encapsulated dye

m_{di}- mass of feeding dye

EE= 38.51%

Dye Loading (DL)

$$DL = \frac{m_{ed}}{(m_{ed} + m_p)} x100\%$$

m_p-mass of polymer

DL= 7.1 %

Nanoparticle characterization

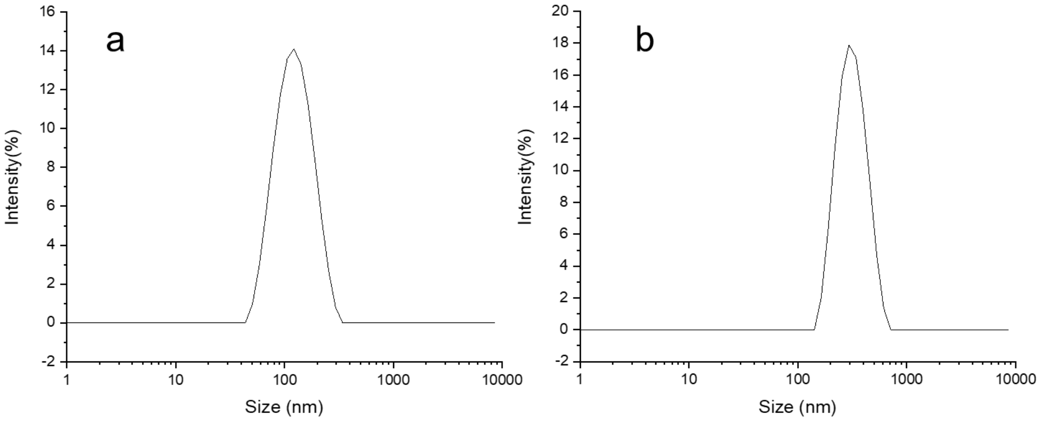

Figure S8. Particle size distribution using dynamic light scattering. a) **TRPZ-bisMPA** NPs b) **TRPZ-127** NPs.

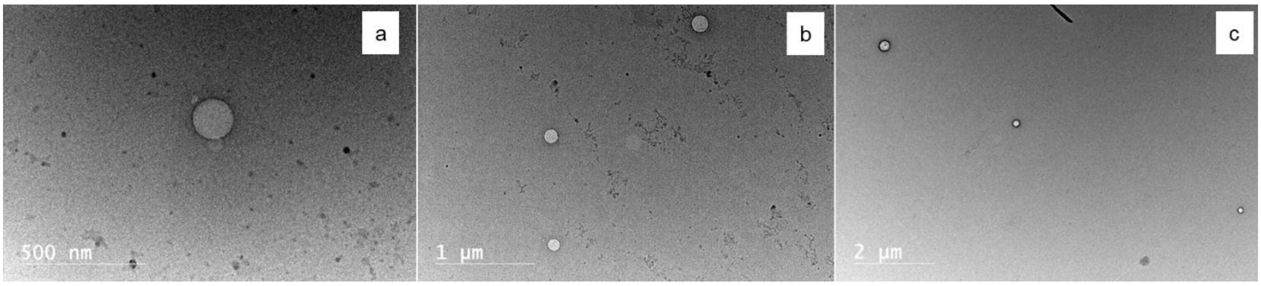

Figure S9. Particle size distribution using dynamic light scattering. a) **TRPZ-bisMPA** NPs b) **TRPZ-127** NPs.

Table S1. Comparison of particle size values for the average hydrodynamic diameter of the nanoparticle systems by TEM and DLS (in number and intensity), and surface charge.

Sample	Nano particle preparation method	R_{TEM} (nm)	R_{hDLS}(nm)	PDI	ζ-potential (mV)
			Number		
TRPZ-bisMPA NPs	Nano precipitation	155.1 ±16	129.9 ± 20.0	0.19	17.2
TRPZ-127 NPs	Nanoprecipitation	181.5± 66	323.5 ± 97.0	0.19	-0.5

Computational details

Gaussian 2009 has been utilized to obtain optimized structures. All structures have been optimized using B3LYP functional with 6-311G(d,p) basis set. Bond order calculation has been carried out using MultiWFN package

HOMO -0.08731

LUMO -0.19355

GAP = 2.45 eV

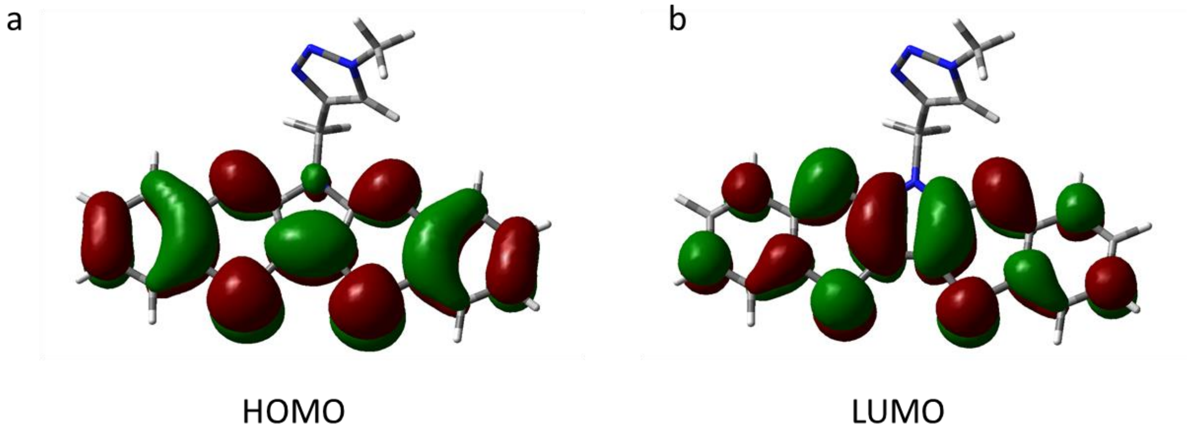

Figure S10. Calculated a) HOMO and b) LUMO energy for TRPZ-bisMPA using B3LYP functional with 6-311G(d,p) basis set.

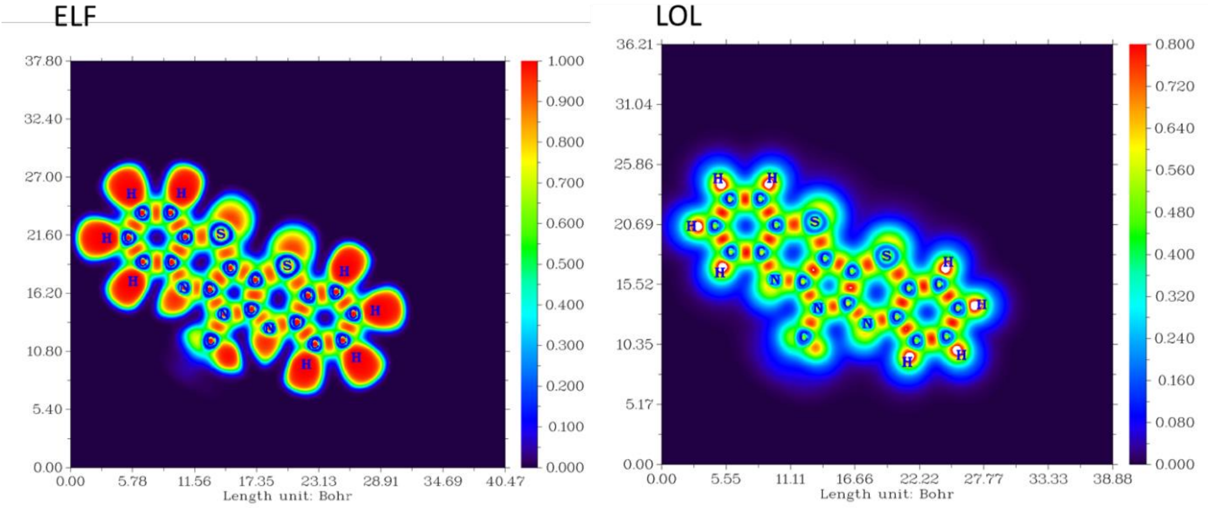

Figure S11. ELF color filled map (left) LOL color filled map (right)

ELF-pi (counting from the left)

Ring 3 0.74

Ring 2 0.86

Ring 1 0.90

HOMA index

Ring 1 0.95

Ring 2 0.40

Ring 3 0.30

Ring 4 and 5 are like rings 1 and 2 respectively.

Energy (cm-1)	Wavelength (nm)	Osc. Strength	Symmetry	Major contributions	Minor contributions
22127.8207596	451.919784991	0.4635	Singlet-A	HOMO->LUMO (95%)	H-1->LUMO (3%)
27374.4573203	365.30404541	0.1979	Singlet-A	H-1->LUMO (92%)	HOMO->LUMO (3%), HOMO->L+2 (3%)
30549.8621072	327.333719704	0.0077	Singlet-A	H-2->LUMO (30%), HOMO->L+1 (66%)	
32065.3778793	311.86284589	0.0536	Singlet-A	H-2->LUMO (46%), HOMO->L+1 (21%), HOMO->L+3 (17%)	HOMO->L+5 (8%)
32387.9996509	308.756332833	0.0064	Singlet-A	HOMO->L+5 (83%)	H-2->LUMO (5%), HOMO->L+1 (3%), HOMO->L+6 (4%)
32546.8908734	307.249010017	0.0696	Singlet-A	H-3->LUMO (49%), HOMO->L+2 (43%)	
32793.6965287	304.936651202	0.118	Singlet-A	H-3->LUMO (43%), HOMO->L+2 (44%)	H-6->LUMO (2%)
33489.7530009	298.598798257	0.0409	Singlet-A	H-4->LUMO (20%), H-2->LUMO (15%), HOMO->L+3 (56%)	HOMO->L+1 (4%), HOMO->L+2 (2%)
33701.0702613	296.726481458	0.0028	Singlet-A	H-6->LUMO (90%)	H-5->LUMO (4%), H-3->LUMO (4%)
34279.3697869	291.720648955	0.0043	Singlet-A	H-4->LUMO (28%), HOMO->L+3 (13%), HOMO->L+4 (49%)	
34897.1904795	286.556019627	0.0002	Singlet-A	H-4->LUMO (39%), HOMO->L+4 (39%)	H-1->L+1 (4%), HOMO->L+3 (6%)
35288.3693776	283.37948668	0.0007	Singlet-A	H-5->LUMO (82%)	H-9->LUMO (2%), H-8->LUMO (8%), H-6->LUMO (4%)

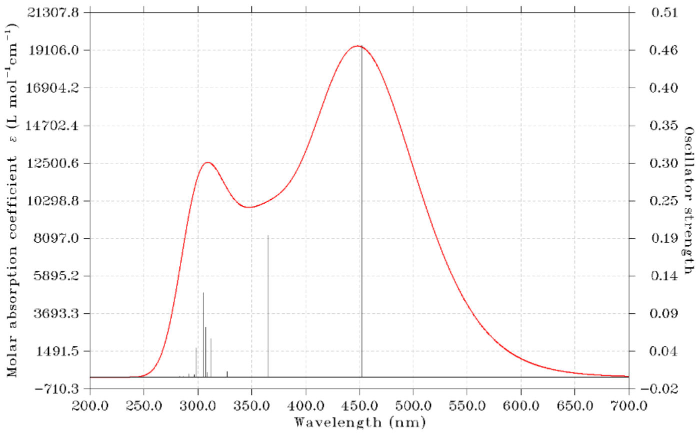

Figure S12. Supporting data for simulated absorbance spectrum

LBO (Laplacian Bond Order)

#	1:	1(S)	3(C)	1.01404309
#	2:	1(S)	14(C)	1.08750825
#	3:	1(S)	16(C)	0.05030342
#	4:	2(N)	4(C)	1.25069294
#	5:	2(N)	13(C)	1.68441280
#	6:	3(C)	4(C)	1.23188457
#	7:	3(C)	6(C)	0.06745094
#	8:	3(C)	8(C)	1.40365830
#	9:	4(C)	5(C)	1.38161208
#	10:	4(C)	7(C)	0.06462780
#	11:	5(C)	6(C)	1.46838876
#	12:	5(C)	8(C)	0.07953179
#	13:	5(C)	9(H)	0.95463008
#	14:	6(C)	7(C)	1.39734846
#	15:	6(C)	10(H)	0.96800138
#	16:	7(C)	8(C)	1.46414501
#	17:	7(C)	11(H)	0.96712185
#	18:	8(C)	12(H)	0.95775849
#	19:	13(C)	14(C)	0.99698981
#	20:	13(C)	15(N)	1.08459828
#	21:	13(C)	17(C)	-0.05260874
#	22:	14(C)	16(C)	1.62604206
#	23:	15(N)	17(C)	1.10185022
#	24:	15(N)	30(C)	0.93901629
#	25:	16(C)	17(C)	1.01131282
#	26:	16(C)	19(S)	1.07692529
#	27:	17(C)	18(N)	1.63063924
#	28:	18(N)	20(C)	1.23713965
#	29:	19(S)	21(C)	1.01418662
#	30:	20(C)	21(C)	1.23876376
#	31:	20(C)	23(C)	0.06523685
#	32:	20(C)	25(C)	1.38011413
#	33:	21(C)	22(C)	1.40738549
#	34:	21(C)	24(C)	0.06806795
#	35:	22(C)	23(C)	1.46144801
#	36:	22(C)	25(C)	0.07854932
#	37:	22(C)	26(H)	0.95760220
#	38:	23(C)	24(C)	1.40012194
#	39:	23(C)	27(H)	0.96713039
#	40:	24(C)	25(C)	1.46568536
#	41:	24(C)	28(H)	0.96820205
#	42:	25(C)	29(H)	0.95768803
#	43:	30(C)	31(H)	0.95536863
#	44:	30(C)	32(H)	0.93791886
#	45:	30(C)	33(C)	0.99376554
#	46:	33(C)	34(N)	1.42718375
#	47:	33(C)	36(N)	0.05263433
#	48:	33(C)	37(C)	1.45356042
#	49:	34(N)	35(N)	1.44160994
#	50:	34(N)	36(N)	0.08833933
#	51:	35(N)	36(N)	1.16609454
#	52:	35(N)	37(C)	0.11555689
#	53:	36(N)	37(C)	1.17799705
#	54:	36(N)	39(C)	0.91166959
#	55:	37(C)	38(H)	0.95222450
#	56:	39(C)	40(H)	0.96303163
#	57:	39(C)	41(H)	0.96353337
#	58:	39(C)	42(H)	0.95811403

Mayer bond order analysis

Total valences and free valences defined by Mayer:

Atom	1(S) :	2.27986780	0.00000000
Atom	2(N) :	3.17168555	0.00000000
Atom	3(C) :	3.73938547	0.00000000
Atom	4(C) :	3.99615677	0.00000000
Atom	5(C) :	3.90483844	0.00000000
Atom	6(C) :	3.90674352	0.00000000
Atom	7(C) :	3.91889563	0.00000000
Atom	8(C) :	3.96997993	0.00000000
Atom	9(H) :	0.99104450	0.00000000
Atom	10(H) :	0.97211988	0.00000000
Atom	11(H) :	0.97276859	0.00000000
Atom	12(H) :	0.97877087	0.00000000
Atom	13(C) :	3.79721714	0.00000000
Atom	14(C) :	3.90659066	0.00000000
Atom	15(N) :	3.28433361	0.00000000
Atom	16(C) :	3.90961518	0.00000000
Atom	17(C) :	3.77618851	0.00000000
Atom	18(N) :	3.12438396	0.00000000
Atom	19(S) :	2.26812517	0.00000000
Atom	20(C) :	3.98409987	0.00000000
Atom	21(C) :	3.75053841	0.00000000
Atom	22(C) :	3.97169545	0.00000000
Atom	23(C) :	3.91767148	0.00000000
Atom	24(C) :	3.90927670	0.00000000
Atom	25(C) :	3.90573970	0.00000000
Atom	26(H) :	0.97862468	0.00000000
Atom	27(H) :	0.97285912	0.00000000
Atom	28(H) :	0.97247652	0.00000000
Atom	29(H) :	0.99134206	0.00000000
Atom	30(C) :	3.81762498	0.00000000
Atom	31(H) :	0.97028520	0.00000000
Atom	32(H) :	1.00330184	0.00000000
Atom	33(C) :	3.91903291	0.00000000
Atom	34(N) :	3.01194875	0.00000000
Atom	35(N) :	2.78914912	0.00000000
Atom	36(N) :	3.39535466	0.00000000
Atom	37(C) :	3.71474061	0.00000000
Atom	38(H) :	0.99991913	0.00000000
Atom	39(C) :	3.80194027	0.00000000
Atom	40(H) :	0.95952824	0.00000000
Atom	41(H) :	0.95998572	0.00000000
Atom	42(H) :	0.98898668	0.00000000

Photophysical characterization

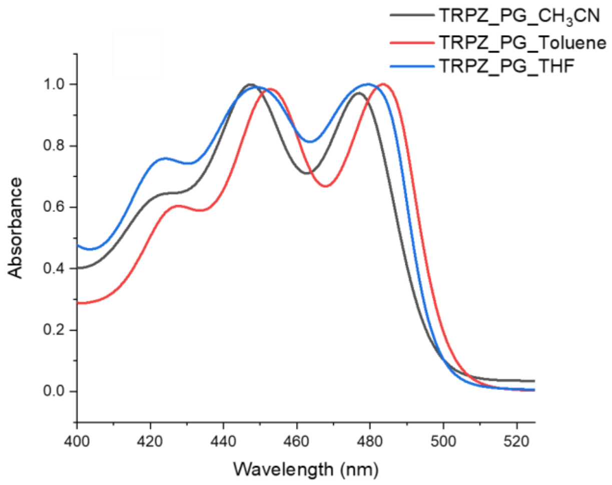

Figure S13. Solvatochromism of the TRPZ-PG dye in acetonitrile, toluene, THF; Less polar excited state relative to ground states as indicated by blue-shifted spectra with increase polar media acetonitrile (polarity index 5.8, dielectric constant 37.5); THF (polarity index 4.0, dielectric constant 7.58); toluene (polarity index 2.4, dielectric constant 2.38)

Fluorescence lifetime

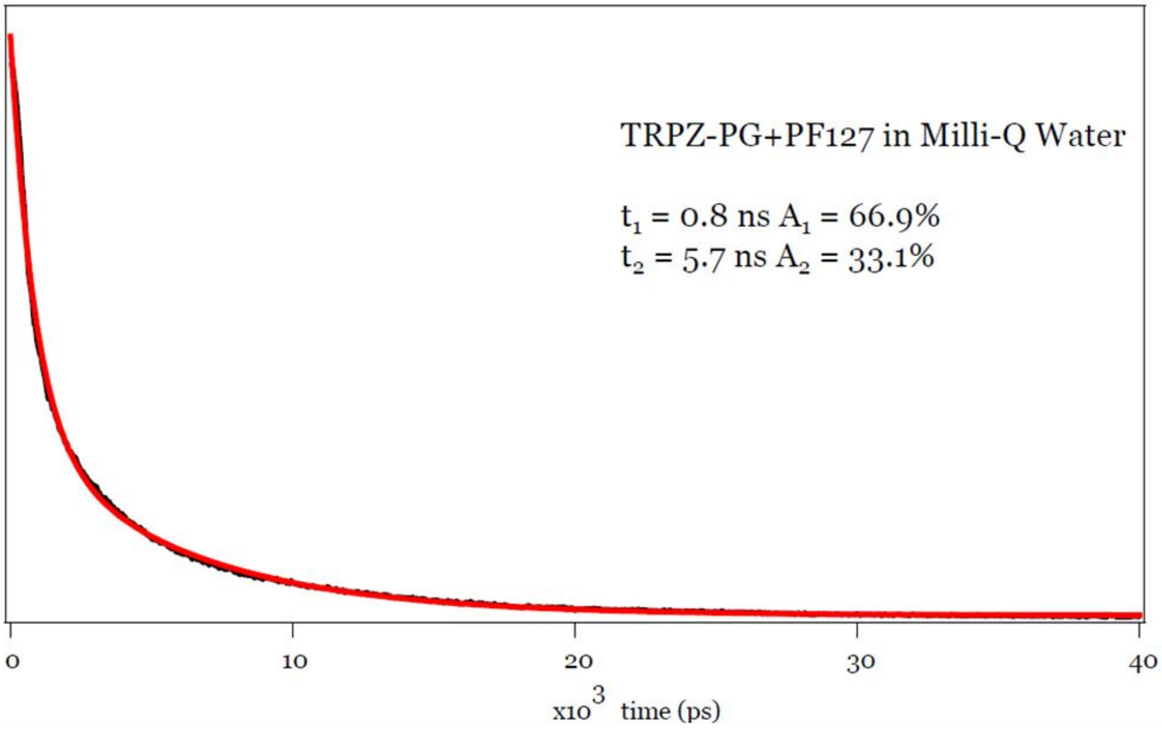

Figure S14. Time-correlated single photon-counting (TCSPC) fluorescent decay curve for TRPZ-PG in THF.

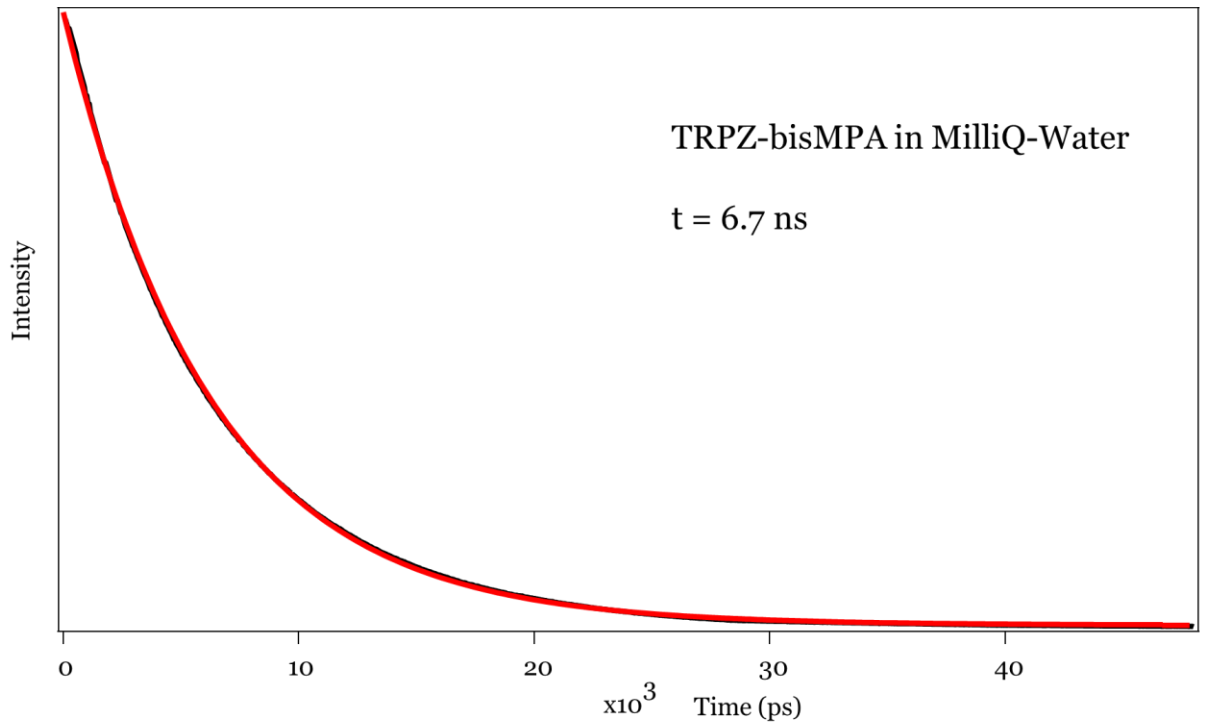

Figure S15. Time-correlated single photon-counting (TCSPC) fluorescent decay curve for TRPZ-127 NPs in Milli-Q Water.

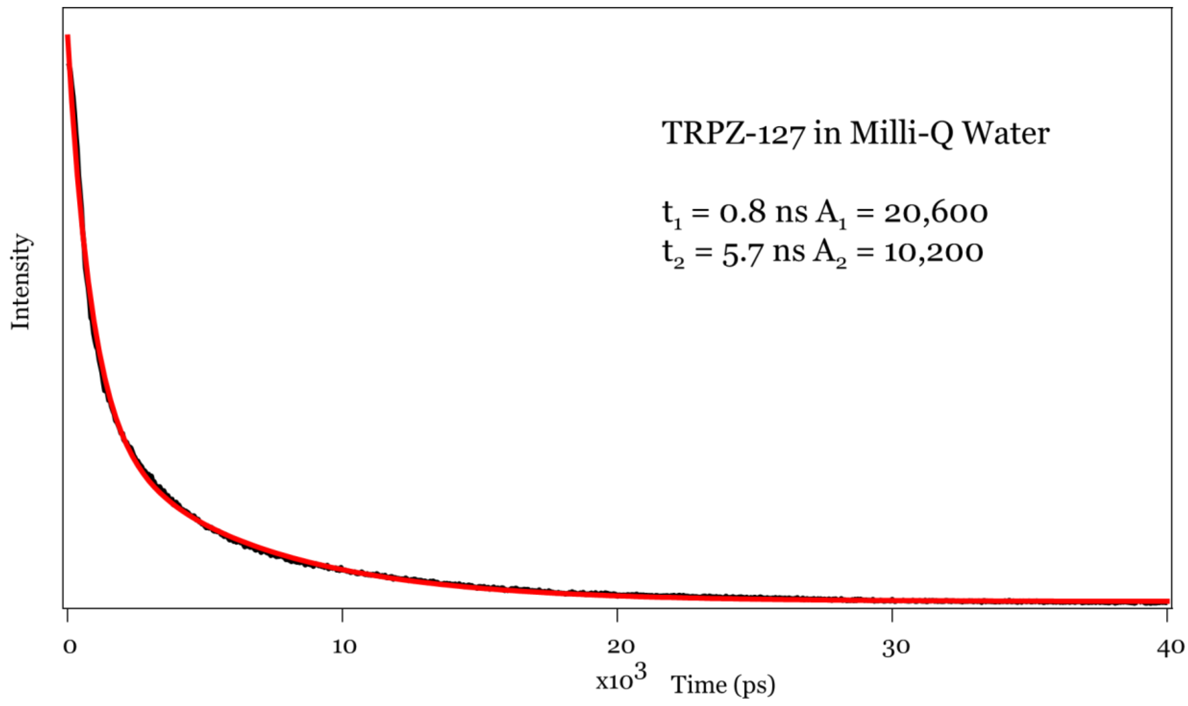

Figure S16. Time-correlated single photon-counting (TCSPC) fluorescent decay curve for TRPZ-bisMPA NPs in Milli-Q Water.

Cellular Viability, Uptake, and Imaging

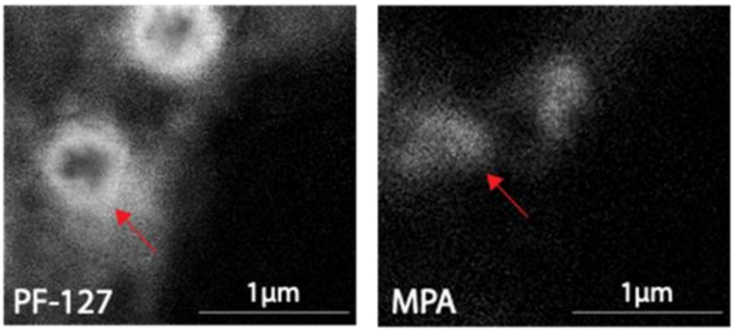

Figure S17. Stimulated emission depletion microscopy (STED)- super-resolution images of TRPZ-127 NPs and TRPZ-bisMPA NPs. (Arrow point shows lysosome).

STED images are shown in Figure S13 where the red arrow points to the lysosome. The image contrast shows that TRPZ-127 NPs are localized to the membrane and the TRPZ-bisMPA NPs are throughout the lysosomes. These results provide further evidence of TRPZ becoming associated within membranes, while TRPZ-bisMPA NPs are distributed uniformly throughout the interior of the lysosome.

References

Dimroth, P., and Reicheneder, F. (1969). Novel Pigments from Dichloromaleimides. *Angewandte Chemie International Edition in English* 8(10)**,** 751-752. doi: https://doi.org/10.1002/anie.196907512.

Wu, Y., Wang, K., Huang, S., Yang, C., and Wang, M. (2017). Near-Infrared Light-Responsive Semiconductor Polymer Composite Hydrogels: Spatial/Temporal-Controlled Release via a Photothermal "Sponge" Effect. *ACS Applied Materials & Interfaces* 9(15)**,** 13602-13610. doi: 10.1021/acsami.7b01016.

Yaddehige, M.L., Chandrasiri, I., Barker, A., Kotha, A.K., Dal Williams, J.S., Simms, B., et al. (2020). Structural and Surface Properties of Polyamidoamine (PAMAM) – Fatty Acid-based Nanoaggregates Derived from Self-assembling Janus Dendrimers. *ChemNanoMat* 6(12)**,** 1833-1842. doi: https://doi.org/10.1002/cnma.202000498.
